# Molecular characterization of norovirus false-positive results on the BioFire FilmArray gastrointestinal panel

**DOI:** 10.1128/jcm.00745-26

**Published:** 2026-07-27

**Authors:** Jonathan C. Reed, Clarice Mauer, Andrew R. Mack, Erez A. Theriault, Giannoula S. Tansarli, Ferric C. Fang, Lori Bourassa, Alexander L. Greninger

**Affiliations:** 1 Department of Laboratory Medicine and Pathology, University of Washington Medical Center, Seattle, Washington, USA; 2 Department of Microbiology, University of Washington Medical Center, Seattle, Washington, USA; 3 Vaccine and Infectious Disease Division, Fred Hutchinson Cancer Research Center 7286, Seattle, Washington, USA; Vanderbilt University Medical Center, Nashville, Tennessee, USA

**Keywords:** gut microbiota, false positive, norovirus, bioMérieux, BioFire, FDA recall

## Abstract

Molecular syndromic panels such as the BioFire FilmArray Gastrointestinal Panel (BF-GIP) have been widely adopted for gastrointestinal illness diagnosis due to their fast turnaround times and broad pathogen coverage. Recently, the BF-GIP demonstrated increased rates of norovirus false-positive detections, prompting a Class II recall of more than two million tests in February 2024 and resulting in an estimated burden of more than a quarter-million false-positive norovirus results reported over the last 5 years. We examined the prevalence of BF-GIP norovirus false positives across four hospitals from December 2024 to June 2025. Among 185 BF-GIP norovirus-positive results confirmed with the BD MAX Enteric Viral Panel, the false discovery rate ranged from 31% to 74% across sites, with the highest rate seen at a specialized cancer care hospital. Deep sequencing of BF-GIP pouches (*n* = 42) confirmed the Noro-1 assay as the primary source of off-target amplification, identifying 78 off-target species—predominantly commensal stool bacteria—compared to only two species for the Noro-2 assay. Off-target species amplified by the Noro-1 assay were recovered from both false-positive and true-negative pouches, suggesting no single species accounted for the false-positive results. Partial primer complementarity at off-target loci and amplicon Tm values within the acceptable range support mispriming of gut microbiota as the underlying cause. False-positive pouches exhibited significantly higher crossing point values than true-positive pouches for both assays (Noro-1: 26.6 vs 11.1, *P* = 0.013; Noro-2: 30.0 vs 13.1, *P* < 0.001), consistent with low-level off-target amplification. These findings highlight the high false discovery rate of the Noro-1 assay, identify bacterial species involved in mispriming, and demonstrate the need to redesign this assay to ensure reliable testing and improved patient care.

**IMPORTANCE:**

Syndromic molecular panels have revolutionized gastrointestinal diagnostics. However, recent data have suggested significant norovirus false-positive results associated with the BioFire FilmArray Gastrointestinal Panel. Here, we investigate a major diagnostic failure associated with the 2024 Class II recall of this assay, revealing norovirus false discovery rates as high as 74% in certain clinical settings. By deep sequencing amplicons from FilmArray pouches, we identified widespread cross-reactivity of the Noro-1 assay with stool microbiome nucleic acid. Off-target Noro-1 amplicons were detected from 78 bacterial species across 42 clinical pouches. For 16 species with the highest read counts per pouch, amplicons mapped to discrete genomic loci with partial primer overlap, consistent with mispriming. The identification of these discrete loci, combined with the repeatedly high false discovery rate reported across multiple studies, creates a clinical imperative to redesign this assay. Our work also highlights the ongoing need for rigorous post-market surveillance and the utility of deep sequencing in troubleshooting diagnostic assay failures.

## INTRODUCTION

Diarrheal diseases are a major public health issue and one of the leading causes of global child mortality (1). Norovirus, a positive-sense, non-enveloped RNA virus, is the leading cause of acute gastroenteritis and foodborne illness worldwide, accounting for roughly 685 million cases globally (2) and approximately 109,000 hospitalizations and nearly $600 million in medical charges each year in the United States (3).

Of the 10 known norovirus genogroups, five (GI, GII, GIV, GVIII, and GIX) cause disease in humans, with the vast majority of human infections due to GII viruses (4).

Diagnosis of norovirus and other causes of infectious gastroenteritis in clinical laboratories is increasingly performed using multiplexed molecular panels, which generally provide rapid results with high sensitivity and specificity (5). A commonly used molecular panel for gastrointestinal illness is bioMérieux’s BioFire FilmArray Gastrointestinal Panel (BF-GIP)—a sample-to-answer assay that isolates, amplifies, and detects nucleic acid from 22 gastrointestinal pathogens, including norovirus (6). Within the panel, pathogen detection relies on a DNA-binding dye-based qPCR, and norovirus is identified through assays separately targeting the two main genogroups that cause disease in humans, GI (Noro-1) and GII (Noro-2) (6). In addition, the assay detects GIX.1 noroviruses. Detection with either or both the Noro-1 and Noro-2 assays produces a Norovirus GI/GII Detected result. BF-GIP use has expanded rapidly, with data in BioFire Syndromic Trends (a cloud-based database of FilmArray results) showing a nearly 10-fold increase in the total number of tests performed from 2016 to 2018 (7, 8), though BF-GIP test volume data are not publicly disclosed by bioMérieux.

Beginning around 2021, an elevated incidence of norovirus false-positive results has been reported on the BF-GIP platform. Despite widespread recognition of this issue among infectious disease providers and clinical microbiologists, only four academic studies have been published to date. Caza et al. found 27.4% of BF-GIP norovirus positives from February 2023 to May 2024 could not be confirmed using a second molecular method (9). Matic et al. reflexed 50 BF-GIP positives to the Xpert Norovirus test from October 2023 to January 2024 and found 36% could not be confirmed (10). A majority (16/18) of these false-positive samples had atypical multimodal melting curves for the BF-GIP Noro-1 target (10). Yoo et al. analyzed 246 stool specimens tested on the BF-GIP, Kogene PowerChek, and BD MAX platforms between May 2023 and January 2024 and observed a 26% false discovery rate (FDR) on the BF-GIP (11). They also noted significantly higher norovirus crossing point (Cp) values in false positives compared to true-positive specimens, with most false positives also displaying atypical melting curves. More recently, Daley et al. found that 66.2% of BF-GIP norovirus positives from May 2024 to December 2025 were false-positive results (12).

In May 2023, bioMérieux launched an internal investigation and conducted a postmarket performance follow-up study, finding that the negative percent agreement (NPA) of the norovirus assay declined from 98.8% in the original clinical study to 96.5% in the postmarket study. Although not directly reported, the false discovery rates calculated from these data increased from 25.7% in the original clinical study to 46.0% in the postmarket study (13, 14). To address this, bioMérieux initiated a Class II recall of more than 2.3 million BF-GIP tests and recommended confirmatory testing when norovirus results are discordant with clinical presentation. However, the assay itself has not been modified to date, and the requirement for reflex testing introduces additional costs and diagnostic delays for the most common cause of acute gastroenteritis worldwide (15).

Here, we examined norovirus false discovery rates associated with BF-GIP testing at our medical center, using the BD MAX Enteric Viral Panel for confirmatory testing. To investigate the basis of these false positives, we deep sequenced BF-GIP amplicons from testing of clinical stool specimens and determined the off-target templates amplified by the norovirus BF-GIP assay.

## MATERIALS AND METHODS

### BF-GIP testing

Stool was collected from patients with gastroenteritis presenting to either UW Medical Center (UWMC; Academic Tertiary Care Hospital), UW Medical Center—Northwest (NW; Community Hospital), Fred Hutchinson Cancer Center/Seattle Cancer Care Alliance (SCCA; Specialized Cancer Center), or Harborview Medical Center (HMC; Public County Hospital). Stool was transferred to Cary-Blair media and tested by the BF-GIP (bioMérieux, RFIT-ASY-0116). BF-GIP testing from UWMC, NW, and SCCA was performed at the UWMC Microbiology Lab, while BF-GIP testing from HMC was performed at HMC. Norovirus-positive results from BF-GIP were confirmed at the UWMC with the BD MAX Enteric Viral Panel (BD, 443985), which was internally validated for norovirus detection by UWMC Microbiology in 2024. After testing, BF-GIP pouches were stored at 4°C for a median of 5 days (interquartile range 3–11) prior to library preparation and deep sequencing.

### Collection of BF-GIP pouches, library preparation, sequencing, and read processing

To investigate the cause of the norovirus-positive results, we obtained 42 BF-GIP pouches from UWMC Microbiology (28 norovirus-positive and 14 norovirus-negative as reported by the BioFire instrument). To infer BF-GIP assay primer sequences, we also obtained three control pouches tested with the Microbiologics Gastrointestinal Control materials (catalog no. 8236): Panel 1 (MC1), Panel 2 (MC2), and negative (Neg). Finally, two additional clinical pouches, one *Campylobacter*-positive and one enteroaggregative *Escherichia coli* (EAEC)-positive, were used to infer primers for assay targets not detected in MC1 or MC2. All pouches were subjected to two independent library preparations and sequencing, with six discrepant pouches prepared a third time and the two most concordant preparations selected for the final analysis.

Amplicons were obtained from BF-GIP pouches by separating the array from the pouch with a clean razor blade and using a P20 pipette to remove ~20 µL of fluid from the FilmArray input channel. Sequencing libraries were generated from collected amplicons using a KAPA HyperPlus Kit (Roche, KK8515). End repair and dA-tailing were performed per the manufacturer’s instructions, with 1 µL of uncleaned amplicons diluted to 35 µL with 5 mM Tris-HCl pH 8.0 as the DNA input, molecular grade water substituted for the KAPA Frag enzyme, and the fragmentation incubation omitted. Adapter ligation was performed per the manufacturer’s instructions, with 5 µL 750 nM Y-stub iTru adapter stock (Integrated DNA Technologies, Table S1) (16) substituted for KAPA Adapters. Reactions were then cleaned twice with Beckman Coulter Ampure XP Beads (A63882) with a bead-to-sample volume ratio of 1.5, followed by three 80% ethanol washes and final elution into 25 µL of 5 mM Tris-HCl pH 8.0. Full-length, dual-indexed TruSeq-based primers (Table S1) were added by PCR using Platinum Taq High-Fidelity (Invitrogen, 11304029) in a 50 µL reaction containing 20 µL template, 5 µL 10× PCR buffer, 2 µL 50 mM MgSO_4_, 1 µL 10 mM dNTPs, 5 µL 10 µM TruSeq primers, and 0.2 µL polymerase. PCR was performed with an initial denaturation of 98°C for 45 s, 11 cycles of 98°C for 15 s, 60°C for 30 s, and 72°C for 30 s, and a final extension of 72°C for 1 min. Amplicons were cleaned with Ampure XP beads with a bead-to-sample volume ratio of 1.2, followed by two 80% ethanol washes and eluted in 20 µL 5 mM Tris-HCl pH 8.0. Amplicon mass was quantified using Qubit 1× HS DNA Assay (Invitrogen, Q33231) and checked by gel electrophoresis prior to pooling and sequencing on 2 × 150 bp runs on the NextSeq 2000.

Paired-end reads were merged using VSEARCH (version 2.30.0) (17) with staggered merging enabled, a maximum of 10 mismatches permitted, and read-through adapter sequence trimmed. Merged reads were deduplicated with read counts annotated and both orientations collapsed, then converted to FASTA format for BLASTn submission.

### BLASTn search and taxonomic assignment

For BLASTn analysis of the MC1 and MC2 control pouches, FASTA query files were filtered to remove abundant *Schizosaccharomyces pombe* RNA process control reads using a custom bash script that identified reads containing the inferred *S. pombe* primer sequences in either orientation without a positional requirement. Filtered sequence sets were then subjected to BLASTn and taxonomic assignment. For BLASTn analyses of the Noro-1 and Noro-2 assays, sequence sets were first filtered for Noro-1 and Noro-2 assay amplicons using a custom bash script that identified reads containing the inferred primer sequences in either orientation without positional requirements. Filtered sets were further processed using a custom R script with the Biostrings (version 2.76.0) and ShortRead (version 1.66.0) R packages (18, 19) to validate primer sites and trim primer sequences. Primer sites were considered valid if detected sites were within 1 bp of the read ends, there were at least 10 bp between sites, only a single pair of sites was detected, and the sites were present in the correct orientation for amplification. Processed FASTA query files were submitted to BLASTn (version 2.17.0+) via Nextflow (version 25.04.6) against the NCBI Core nt database (downloaded on 22 August 2025) configured to return a maximum of five aligned sequences per query and all High-scoring Segment Pairs for any single query-subject pair, and with a word size of 11.

Taxonomic designations from species to order level were retrieved for each BLASTn hit using the R package taxize (version 0.10.0) (20), with taxonomy IDs obtained from accession numbers via rentrez (version 1.2.4) (21). Results were filtered to retain only hits from queries with at least five merged reads, query coverage exceeding 65%, and an *E*-value below 1 × 10^−5^. For each query, only the highest bitscore alignment with the highest percent identity was retained. Hits with identical bitscore and percent identity aligning to the same species were collapsed, while those aligning to different species were assigned the highest resolved taxonomic level (e.g., unresolved *Lachnospiraceae*). Taxonomic assignments were only considered valid if they appeared in both independent library preparations.

Taxonomically unresolved queries were grouped by 95% sequence identity using BBTools dedupe.sh (v39.81b), and a representative sequence from each group was subjected to megablast search of the whole-genome shotgun (WGS) database (accessed on 30 March 2026), restricted to the family-level taxid from the initial BLASTn search. This subset included queries where species-level identification could not be resolved due to multiple hits with identical bitscore and percent identity, or where a single species was identified at below 95% identity. The highest-scoring hit with at least 95% sequence identity was then assigned to all queries within a group. WGS and Core nt results were then combined and reprocessed using the filtering and taxonomic assignment pipeline described above.

Pouches with unresolved species-level assignments exceeding 5% of off-target normalized RPM were reviewed and, where possible, resolved to a single species. [*Clostridium*] *innocuum* accessions assigned to Clostridiaceae in NCBI were manually reassigned to Erysipelotrichaceae based on published phylogenomic reclassification (22). Queries unresolved between [*Clostridium*] *innocuum* and *Erysipelotrichaceae* bacterium I46 were resolved to [*Clostridium*] *innocuum*, as whole-genome alignment confirmed these accessions represent the same organism. Queries unresolved between *Parabacteroides distasonis* (CP103128.1) and *Siphoviridae* sp. ctPAi1 (BK014842.1) were resolved to *Parabacteroides distasonis*, as BLASTn analysis of the 10 kb region surrounding the mapped locus in BK014842.1 showed 99.98% identity to CP103128.1, and the record contains a *Siphoviridae* major capsid protein sequence elsewhere, suggesting a chimeric record representing an integrated prophage. Queries unresolved among *Clostridiales* bacterium (AP018533.1), *Lachnospiraceae* bacterium (AP018536.1), *Faecalibacterium* sp. i25-0019-C1 (AP031428.1), and *Enterocloster bolteae* (CP022464.2) were resolved to *Enterocloster bolteae*, as CP022464.2 represents the complete genome of the ATCC type strain (BAA-613), the only validly named species-level reference among the four accessions, with the remaining accessions representing unnamed organisms not yet formally described at the species level.

### Assessment of *S. pombe* primer specificity

The specificity of the inferred *S. pombe* primer sequences was assessed by screening *S. pombe*-filtered reads for the presence of the expected 66 bp amplicon sequence using vmatchPattern from the Biostrings R package, allowing a maximum of three mismatches in either orientation. The proportion of filtered reads matching the expected amplicon sequence was calculated from deduplicated read counts.

### Inference of BF-GIP assay primers

*S. pombe* RNA process control assay primers were inferred from amplicons from BF-GIP testing of the negative control (Microbiologics, 8236), in which *S. pombe* amplicons were readily identifiable as the dominant reads given the absence of competing template. These reads were mapped to an *S. pombe* reference sequence (NM_001018402.3) using the Geneious mapper at medium-low sensitivity (Geneious Prime 2024.0.7), and 15 nt from each end of the consensus sequence were designated as the inferred primers. A similar approach was used to infer primers for the *aggR* target of the EAEC assay and one of the two *Campylobacter* assays using supplemental EAEC-positive and *Campylobacter*-positive pouches.

Remaining BF-GIP assay primers were inferred by subjecting *S. pombe*-subtracted MC1 and MC2 reads to the BLASTn and taxonomic assignment workflow. Sequences were then taxonomically grouped and aligned using MAFFT (v7.490) (23). Consensus sequences from these alignments were subjected to BLASTn to identify an appropriate reference, and reads were realigned with the identified reference. For targets except Noro-1 and Noro-2, 15 nt from each end of the consensus sequence were designated as the forward and reverse primers, with degeneracies allowed. Noro-1 primers consisted of a 17-nucleotide sequence with a sixfold degeneracy and a 22-nucleotide sequence with no degeneracies. Noro-2 primers consisted of a 14-nucleotide primer with a 16-fold degeneracy and a 15-nucleotide primer with a 216-fold degeneracy.

To confirm that the inferred Noro-1 and Noro-2 assay primers capture norovirus amplicons from clinical specimens, reads from a true-positive pouch for each genogroup (norovirus GI: pouch 929; norovirus GII: pouch 432) were mapped to pouch-specific reference sequences identified by BLASTn (norovirus GI: KP407450.1; norovirus GII: PQ336852.1) using the Geneious aligner at medium-low sensitivity. The proportion of norovirus-mapped reads captured by the inferred Noro-1 and Noro-2 primer criteria was then calculated.

### Determination of amplicon Tm values and estimates of BF-GIP acceptable Tm ranges

BF-GIP acceptable Tm ranges for the Noro-1 and Noro-2 assays were estimated from pouch results exported from BioFire FireWorks software (version 8), retaining only results with a single instrument-reported Tm value to avoid ambiguity from multimodal melting curves. The minimum and maximum Tm values from this filtered data set were used to define the lower and upper bounds of the estimated acceptable Tm range. Since the instrument reports a positive result based solely on amplicon Tm regardless of target specificity, confirmed true-positive results were not required for this estimate; the Noro-1 range was based on 21 pouches (11 false positive, 2 true positive, 1 control, and 7 supplemental true positive) and the Noro-2 range on 34 pouches (5 false positive, 2 true positive, 1 control, and 26 supplemental true positive). Amplicon Tm values were estimated using the TmCalculator R package (version 1.0.3) (24), with monovalent concentrations of 50 mM, divalent concentrations of 0.85 mM, and the DNA_NN4 nearest-neighbor thermodynamic table (SantaLucia, 2004), conditions selected as they best recapitulated the midpoint of the estimated acceptable range when applied to the expected Noro-1 and Noro-2 amplicon sequences.

### Genome mapping and primer binding site characterization of Noro-1 off-target amplicons

To map off-target amplicons to their source genomes, processed reads from all pouches were pooled, filtered to retain only those with at least 95% BLASTn identity and a resolved species-level identification, and clustered using the cluster_fast algorithm in VSEARCH (version 2.30.4) at 95% sequence identity. Representative sequences comprising at least 10% of total reads for each species were subjected to Microbial Nucleotide BLAST against the complete genomes database with bacteria as the organism filter and megablast optimizations, except for *Enterocloster clostridioformis*, for which the NCBI WGS database was used with *Enterocloster* as the organism filter due to the absence of a suitable reference genome in NCBI Core nt. The highest quality genome-length match was downloaded from NCBI using the Biopython Entrez interface (version 1.86). Reads were aligned to the selected reference sequence using BWA-MEM (version 0.7.19) with a minimum seed length of 25 bp and re-seeding ratio of 2.0, converted to sorted BAM format, and per-base depth calculated using SAMtools mpileup (version 1.23). Pileup files were visualized using Plotnine (version 0.15.3).

To determine Noro-1 primer overlap with off-target loci, representative sequences were subjected to BLASTn against the NCBI Core nt database using megablast presets, with the highest bitscore and percent identity hit selected as the reference for each species. For *Enterocloster clostridioformis*, the reference identified in the genome mapping analysis described above was used. Reference sequences were downloaded using the Biopython Entrez interface (version 1.86), and BLASTn alignment with primers was used to determine the positioning (using alignment coordinates qstart, qend, sstart, and send) and mismatch content of primer overlap at each locus. The primer overlap figure was generated using DNA Features Viewer (version 3.1.5) and Matplotlib (version 3.10.8).

### Statistical methods

The FDR was calculated as the proportion of BF-GIP norovirus-positive results that were not confirmed by the BD MAX Enteric Viral Panel (i.e., one minus the positive predictive value). FDR is reported here as a measure of test performance in the clinical populations studied and reflects both the analytical specificity of the assay and the pre-test probability of norovirus infection in each setting.

False-positive burden was modeled across two NPA values derived from published and regulatory sources (98.8% and 96.5%) (6, 14). Norovirus prevalence estimates of 4% and 21% were used, reflecting the approximate prevalence observed in the original multicenter and postmarket performance follow-up studies (6, 14) and the positivity range reported in BioFire Syndromic Trends surveillance data (8). Annual testing volume was estimated at approximately 2.3 million, based on the quantity of BF-GIP tests in circulation at the time of the February 2024 FDA recall (15), assuming a 12-month kit shelf life. False positives were calculated as the product of the true negative pool and the false-positive rate (1 − NPA), where the true negative pool was defined as (1 − prevalence) × total tests.

Log_10_-transformed *S. pombe*-normalized RPM for dominant off-target species were compared between norovirus-false-positive and norovirus-true-negative pouches using two-sided Wilcoxon rank-sum tests implemented in the rstatix R package (version 0.7.3) (25), excluding species with fewer than two observations in either group and without correction for multiple comparisons. BioFire instrument-reported Cp values for the Noro-1 and Noro-2 assays were compared between false-positive and true-positive pouches using a two-sided *t*-test implemented in the rstatix. True-positive pouches included the three sequenced in this study and 28 additional BD MAX-confirmed pouches retrieved from BioFire FireWorks software, for a total of 31. Two false-positive pouches lacking a reported Cp (pouches 488 and 478) were excluded, leaving 23 for analysis. When a pouch was positive for both Noro-1 and Noro-2 assays, the assay with the lower Cp was assigned as the primary assay result, as each assay amplifies its target genogroup more efficiently than the other (Fig. S3). *P*-values were plotted using the ggpubr R package (version 0.6.2) (26), and threshold of 0.05 was used to define statistical significance.

## RESULTS

### Assessing false discovery rates from samples received at UWMC

**Fig 1:**
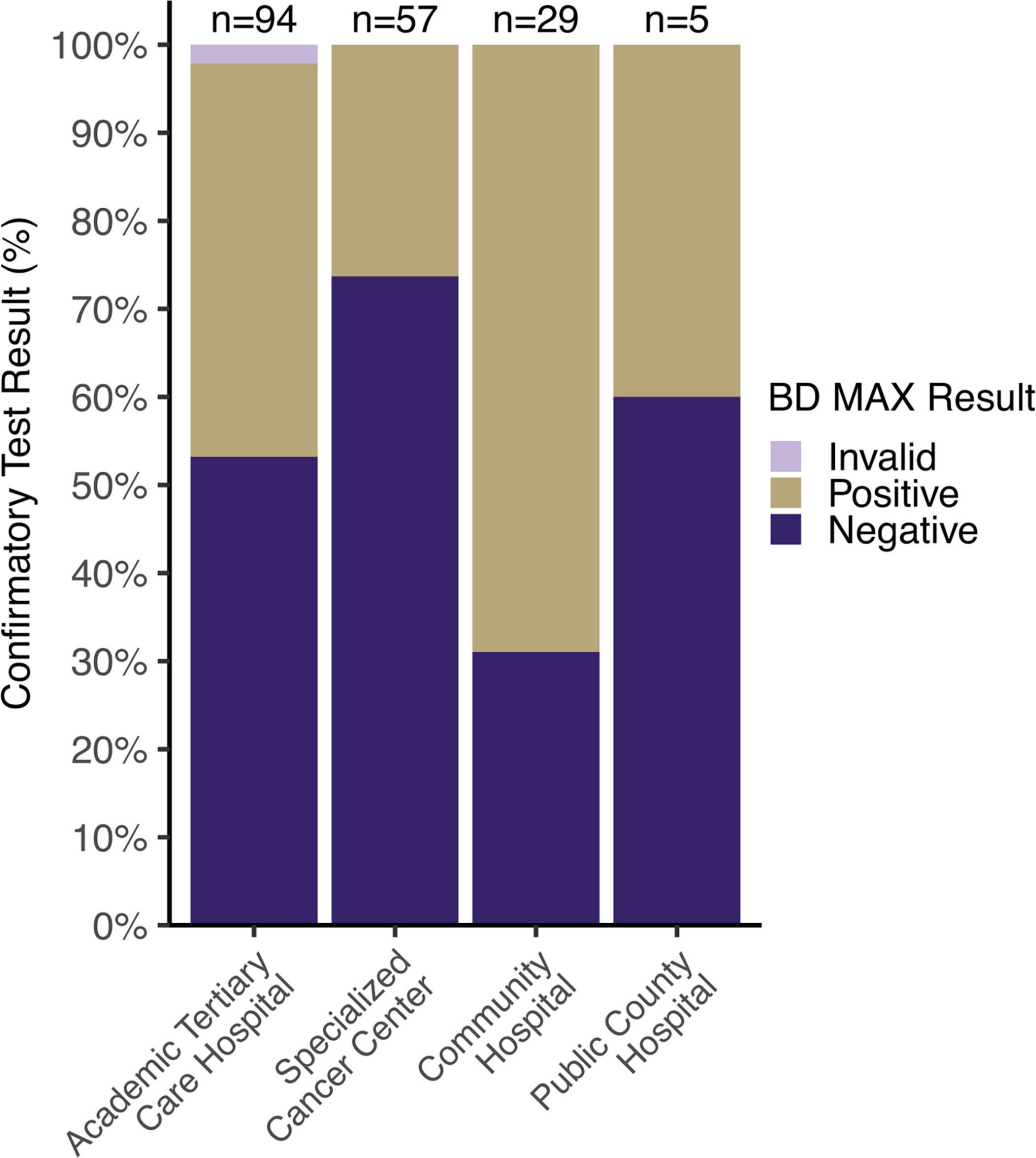
High rates of false-positive BF-GIP norovirus results were identified by confirmatory testing with the BD MAX Enteric Viral Panel across four hospital sites. A total of 185 fecal specimens that tested positive for norovirus by BF-GIP were collected between December 2024 and June 2025 from four hospital sites and reflexed to the BD MAX Enteric Viral Panel for confirmation of norovirus positivity. Reflexed results are shown by site, summarized as the percentage of results that were invalid (light purple), positive (golden), and negative (dark purple). The total number of specimens tested at each site (*n*) is shown above each bar.

To evaluate the BF-GIP norovirus FDR, specimens were collected and tested from December 2024 to June 2025 on the BF-GIP platform from four hospital sites: (i) an academic tertiary care hospital, (ii) a specialized cancer center, (iii) a community hospital, and (iv) a public county hospital. A total of 185 BF-GIP norovirus-positive specimens underwent confirmatory testing with the BD MAX Enteric Viral Panel. Norovirus FDRs varied widely from 31% to 74%, with the academic tertiary care hospital FDR at 53% (*n* = 94), the specialized cancer center FDR at 74% (*n* = 57), the community hospital FDR at 31% (*n* = 29), and the public county hospital FDR at 60% (*n* = 5; Fig. 1).

### Sequencing of norovirus-positive and norovirus-negative BF-GIP pouches

**Fig 2:**
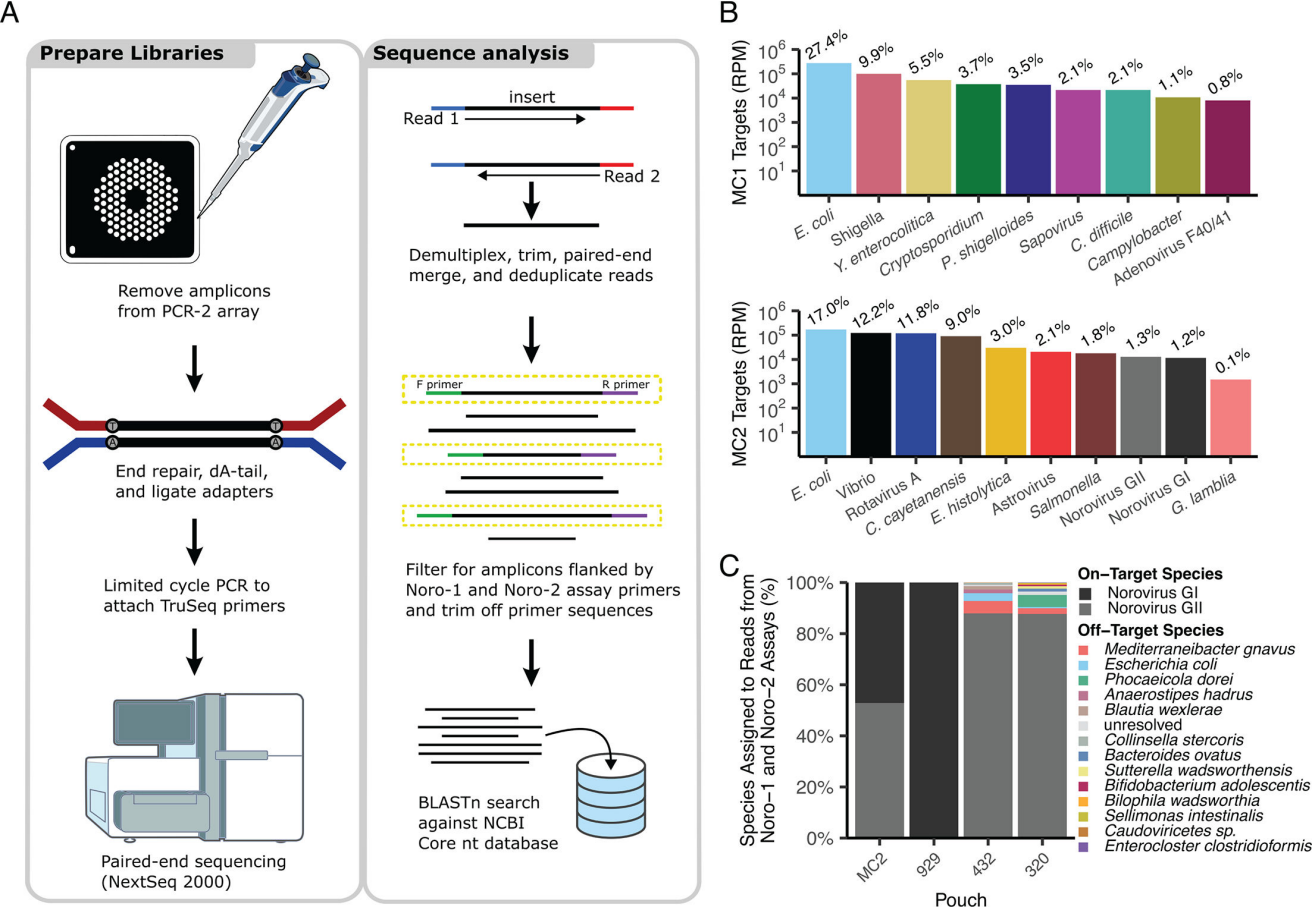
Validation of the BF-GIP amplicon sequencing workflow and identification of Noro-1 and Noro-2 assay primers. (**A**) Workflow for library preparation and sequence analysis of BF-GIP pouch amplicons. (**B**) The Microbiologics Control Panels 1 and 2 (MC1 and MC2), which together contain 22 gastrointestinal pathogens (or surrogate materials; see Table 1), were analyzed by BF-GIP, and the resulting pouch amplicons were sequenced. All reads were subjected to BLASTn search against the NCBI Core nt database, and the log-transformed mean reads per million (RPM; *n* = 2) of each identified target is shown for MC1 (upper panel) and MC2 (lower panel). Related *E. coli* pathotypes were grouped for analysis (see Table 1). The mean percentage of total reads assigned to each target is annotated above each bar. (**C**) The inferred Noro-1 and Noro-2 assay primers identified from the MC2 sequencing data in panel B were applied to filter Noro-1 and Noro-2 amplicons from the MC2 control pouch and three confirmed norovirus-positive clinical specimens (pouches 929, 432, and 320). Filtered amplicons were analyzed by BLASTn against the NCBI Core nt database to determine the species origin of each filtered read. The proportion of reads assigned to each species is shown, with Norovirus GI in black, Norovirus GII in gray, and off-target species in other colors, demonstrating that the inferred primers successfully recover norovirus-specific amplicons from clinical specimens. Pipette and Next Gen Sequencer illustrations are from NIAID NIH BioArt Source (bioart.niaid.nih.gov/bioart/408, bioart.niaid.nih.gov/bioart/386). The database icon is from Bioicons. All three are in the public domain.

To further investigate BF-GIP norovirus false-positive results, we developed an approach to sequence BF-GIP pouch Noro-1 and Noro-2 assay amplicons and identify their taxonomic origin. We obtained 28 BF-GIP pouches with a norovirus-positive result and 14 with a norovirus-negative result. Review of FireWorks results for these 28 norovirus-positive pouches revealed that 21 were positive for the Noro-1 assay alone, 6 were positive for the Noro-2 assay alone, and 1 was positive for both Noro-1 and Noro-2 assays. Of the 28 norovirus-positive BF-GIP pouches, 24 underwent confirmatory testing with the BD MAX Enteric Viral Panel (Fig. S1), of which 22 (91.7%) were confirmed negative (norovirus false positive), while 2 (8.3%) were confirmed positive (norovirus true positive). Confirmatory testing was not available for the remaining four norovirus-positive and 14 norovirus-negative pouches. All 42 pouches were subjected to two independent library preparations and sequencing (Fig. 2A).

### Validation of the BF-GIP amplicon sequencing workflow

To assign sequencing reads to the BF-GIP assays, the primer sequences flanking each assay amplicon had to be inferred. We sequenced pouch amplicons from testing of the Microbiologics Gastrointestinal Control Panels 1 and 2 (MC1 and MC2), which together encompass nearly all BF-GIP targets (Table 1), and the negative control. The *S. pombe* RNA process control assay primers were first inferred from the negative control (see Materials and Methods), and *S. pombe* amplicons were then removed from MC1 and MC2 reads prior to BLASTn identification of the remaining targets, as *S. pombe* reads are compositionally homogeneous. All targets from MC1 and MC2 were successfully identified by our amplicon sequencing workflow (Fig. 2A), with reads per million (RPM) ranging from 7.9 × 10³ to 2.7 × 10⁵ for MC1 and 1.5 × 10³ to 1.7 × 10⁵ for MC2 (Fig. 2B; Table 1). Based on these amplicons, we inferred primers for all but four BF-GIP assays described in the instructions for use (IFU), including the EAEC *aggR* assay, one of the two assays targeting *Campylobacter*, one of the two assays targeting Adenovirus F40/F41, and the enterotoxigenic *E. coli* assay targeting *st1b*. Using supplemental pouches, primers were inferred for two of the four remaining assays, the EAEC *aggR* assay and the second *Campylobacter* assay (see Materials and Methods, Table 1). Primers for the remaining two assays were not inferred in this study. The inferred primers were used to assign reads from all sequenced pouches to specific BF-GIP assays (Fig. S2). As expected, Noro-1 amplicons were detected in all norovirus-positive pouches, and amplicons for other positive panel targets were readily recovered. Unexpectedly, however, Noro-1-derived amplicons were also detected in all norovirus-negative pouches.

**TABLE 1:** List of targets included in the microbiologics control panels and their corresponding BioFire target^*f*^

	Target in MC panel	Group Name^*a*^	BioFire GI panel targets	Alias
MC1	Adenovirus	Adenovirus F40/F41^*b*^	Adenovirus F40/41	*Mastadenovirus faecale*
	*Campylobacter jejuni* ^*c*^	*Campylobacter*	*Campylobacter (C. jejuni/C. coli/C. upsaliensis*)	
	*Clostridioides (Clostridium) difficile*	*C. difficile*	*Clostridioides (Clostridium) difficile* (toxin A/B)	
	*Cryptosporidium parvum*	*Cryptosporidium*	*Cryptosporidium*	
	*Escherichia coli* (EAEC)^*d*^	*E. coli*	Enteroaggregative *E. coli* (EAEC)	
	*Escherichia coli (O157*)	*E. coli*	*E. coli* O157	
	*Escherichia coli (STEC*)	*E. coli*	Shiga-like toxin-producing *E. coli* (STEC) *stx1/stx2*	
	*Plesiomonas shigelloides*	*P. shigelloides*	*Plesiomonas shigelloides*	
	Sapovirus	Sapovirus	Sapovirus (I, II, IV, and V)	
	*Shigella sonnei*	*Shigella*	*Shigella/*Enteroinvasive *E. coli* (EIEC)	
	*Yersinia enterocolitica*	*Y. enterocolitica*	*Yersinia enterocolitica*	
MC2	Astrovirus	Astrovirus	Astrovirus	
	*Cyclospora cayetanensis*	*C. cayetanensis*	*Cyclospora cayetanensis*	
	*Entamoeba histolytica*	*E. histolytica*	*Entamoeba histolytica*	
	*Escherichia coli* (EPEC)	*E. coli*	Enteropathogenic *E. coli* (EPEC)	
	*Escherichia coli* (ETEC)^*e*^	*E. coli*	Enterotoxigenic *E. coli* (ETEC) *lt/st*	
	*Giardia lamblia*	*G. lamblia*	*Giardia lamblia*	*Giardia duodenalis*
	Norovirus GI	Norovirus GI	Norovirus GI/GII	
	Norovirus GII	Norovirus GII		
	Rotavirus A	Rotavirus A	Rotavirus	
	*Salmonella typhimurium*	*Salmonella*	*Salmonella*	
	*Vibrio cholerae*	*Vibrio*	*Vibrio (V. parahaemolyticus/V. vulnificus/V. cholerae*)	

^*a*^ Group name used in Fig. 2B.

^*b*^ The BF-GIP includes a single multiplexed assay for detection of Adenovirus F40 and F41, with presumably separate primer sets for each target. From the MC panels, we were only able to infer primers for the target detecting Adenovirus F40.

^*c*^ The BF-GIP includes two *Campylobacter* assays, but only one was detected in the MC panels. To infer primers for the undetected assay, a BF-GIP *Campylobacter*-positive pouch not included in this study was used and found to contain amplicons to the second *Campylobacter* target (see Materials and Methods).

^*d*^ Surrogate for target was provided in the panel. The BF-GIP includes two EAEC targets, *aggR* and *aatA*, but only *aatA* was detected in the MC panels. To infer primers for *aggR* assay, a BF-GIP EAEC-positive pouch not included in this study was used and found to contain amplicons to the *aggR* target (see Materials and Methods).

^*e*^ The BF-GIP includes three assays for ETEC targeting e*ltA*, *st1a*, and *st1b*. From the MC panels, we were only able to infer primers to *eltA* and *st1a*.

^*f*^ Microbiologics Control Panel 1 (MC1), Microbiologics Control Panel 2 (MC2), and Microbiologics control panel (MC).

For each pouch, Noro-1 and Noro-2 amplicons were subjected to BLASTn to determine whether each pouch contained norovirus-specific sequences, and these results were used in conjunction with BD MAX confirmatory testing to classify pouches as norovirus true positive, false positive, or true negative. Based on this analysis, we identified 25 norovirus false-positive, 3 norovirus true-positive, and 14 norovirus true-negative BF-GIP pouches (Fig. S1).

Among the three norovirus true-positive pouches, Noro-1 and Noro-2 amplicons accounted for 1.5%, 3.6%, and 2.1% of total merged reads for pouches 929, 432, and 320, respectively. Of these, 100% (pouch 929), 87.7% (pouch 432), and 87.4% (pouch 320) were norovirus specific, with all norovirus-specific reads assigned to a single genogroup in each pouch: norovirus GI in pouch 929 and norovirus GII in pouches 432 and 320 (Fig. 2C). A small proportion of off-target amplicons were detected in pouches 432 and 320 (12.1% and 12.3% of total Noro-1 and Noro-2 amplicons, respectively), exclusively associated with the Noro-1 primer set (Fig. S3). In addition, the Noro-1 and Noro-2 primers were not strictly genogroup-specific: 29.7% of norovirus GI amplicons in pouch 929 were captured by the Noro-2 primers, and 23.2% and 21.5% of norovirus GII amplicons in pouches 432 and 320 were captured by the Noro-1 primers (Fig. S3). To confirm that the inferred primers captured most norovirus-specific amplicons from clinical specimens, reads from true-positive pouches 929 and 432 were mapped to pouch-specific norovirus reference sequences identified by BLASTn. The inferred Noro-1 and Noro-2 primers identified 79.5% and 80.6% of norovirus-mapped reads from pouches 929 and 432, respectively, confirming that the inferred primers captured the majority of norovirus-mapped reads from clinical specimens (see Materials and Methods).

As an additional quality control metric, we assessed amplicon recovery across pouches using the internal *S. pombe* RNA process control (Fig. S4). Across all library preparations, 98.8%–99.8% of sequences filtered using the inferred *S. pombe* primers matched the expected target amplicon, confirming inferred *S. pombe* primer specificity. Recovery was highly reproducible between independent library preparations, with fold differences ranging from 1.00- to 1.31-fold for clinical pouches and 1.01- to 1.05-fold for control pouches. Among clinical pouches, mean *S. pombe* recovery ranged from 33.3% to 67.6%, with comparable median values across false-positive, true-negative, and true-positive pouches (45.1%, 48.8%, 54.3%, respectively; Fig. S4), supporting the use of *S. pombe* for cross-pouch normalization within the clinical set. In contrast, *S. pombe* recovery varied substantially among control pouches, with the negative control showing the highest proportion (74.9%) due to the absence of competing templates, while the lower proportions in MC1 and MC2 (27.2% and 21.4%) likely reflect competition from their numerous pathogen templates during either pouch PCR or library preparation. A summary of read counts for each library preparation through each processing step (merging, primer filtering, and BLASTn processing) is provided in Table S2.

### Identification of cross-reactive species in false-positive BF-GIP pouches

**Fig 3:**
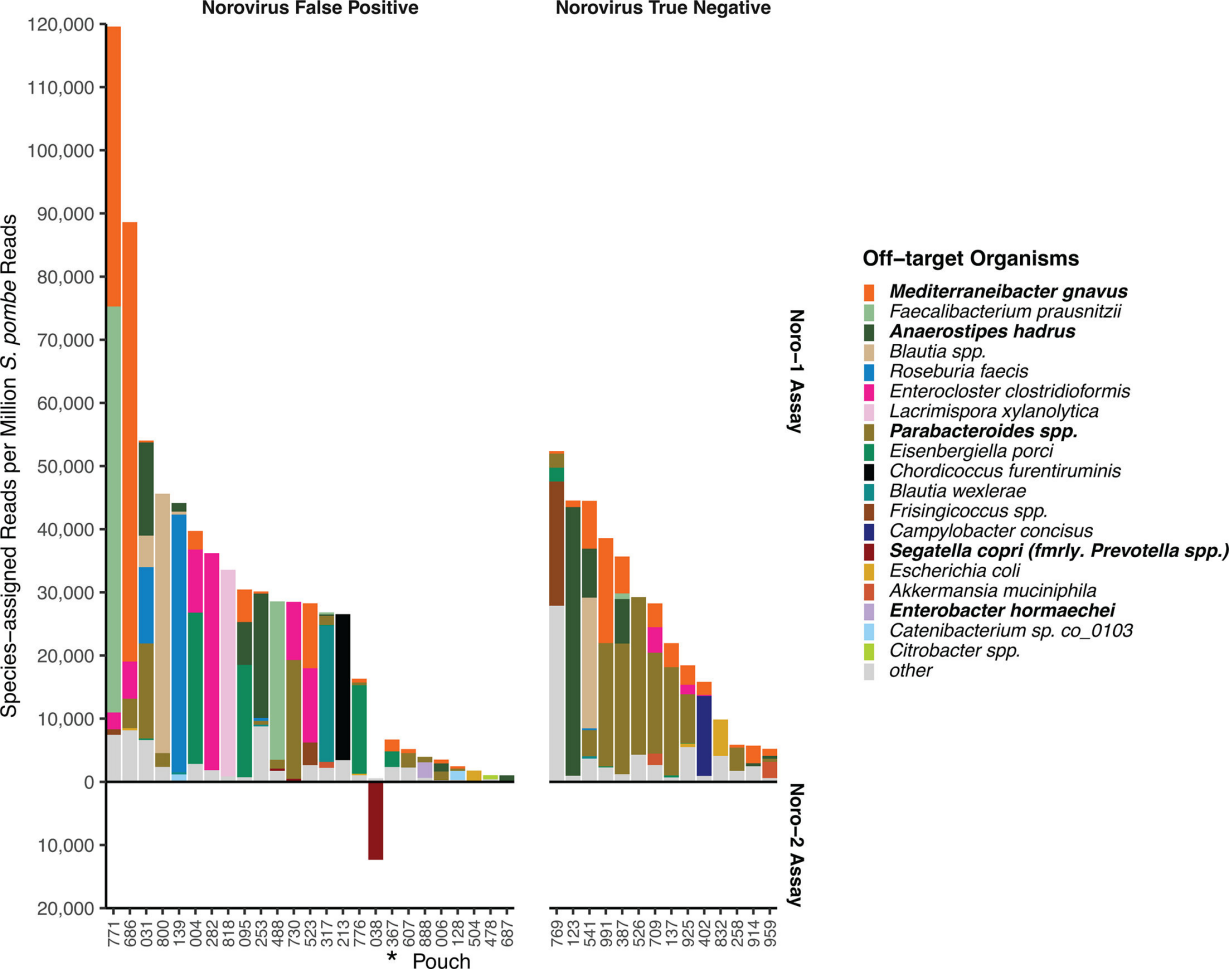
Substantially greater off-target amplification was detected with the Noro-1 assay compared with the Noro-2 assay. Amplicons from norovirus-false-positive (*n* = 25) and norovirus-true-negative (*n* = 14) BF-GIP pouches were independently sequenced in duplicate and analyzed for Noro-1 and Noro-2 amplicons as shown in Fig. 2A. The mean off-target amplicon RPM (*n* = 2 per pouch), normalized to *S. pombe* read count, is shown for the Noro-1 (plotted upward) and Noro-2 (plotted downward) assays. False-positive pouches are shown on the left and norovirus true-negative pouches on the right, ordered by decreasing total off-target amplicon RPM within each group. The dominant off-target organism was identified in each false-positive pouch (based on the highest normalized RPM) and is indicated by fill color. In addition, organisms identified in the bioMérieux IFU are shown in bold. Remaining off-target amplicons are grouped into the other category. Pouch 367 (marked with an asterisk) contained a low-level detectable Noro-2 off-target amplicon from *Alicycliphilus denitrificans* (assigned to the other category) found at 229.9 *S. pombe-*normalized RPM.

Identification of Noro-1 and Noro-2 filtered amplicons from the norovirus false-positive and true-negative pouches revealed a complex pattern of off-target amplification (Fig. 3). Consistent with our findings among true-positive pouches, the Noro-1 assay exhibited substantially higher levels of off-target reads relative to the Noro-2 assay, with higher levels of off-target Noro-1 amplicons detected in 24 of 25 false-positive pouches and all 14 true-negative pouches. In total, 78 resolved off-target species were identified for the Noro-1 assay and 2 for the Noro-2 assay. Queries that could not be resolved to a single species accounted for a small proportion of total off-target Noro-1 normalized RPM in a minority of pouches (5 of 25 false-positive pouches, 0.5%–2.8%, median 1.6%; 1 of 14 true-negative pouches, 0.7%).

**Fig 4:**
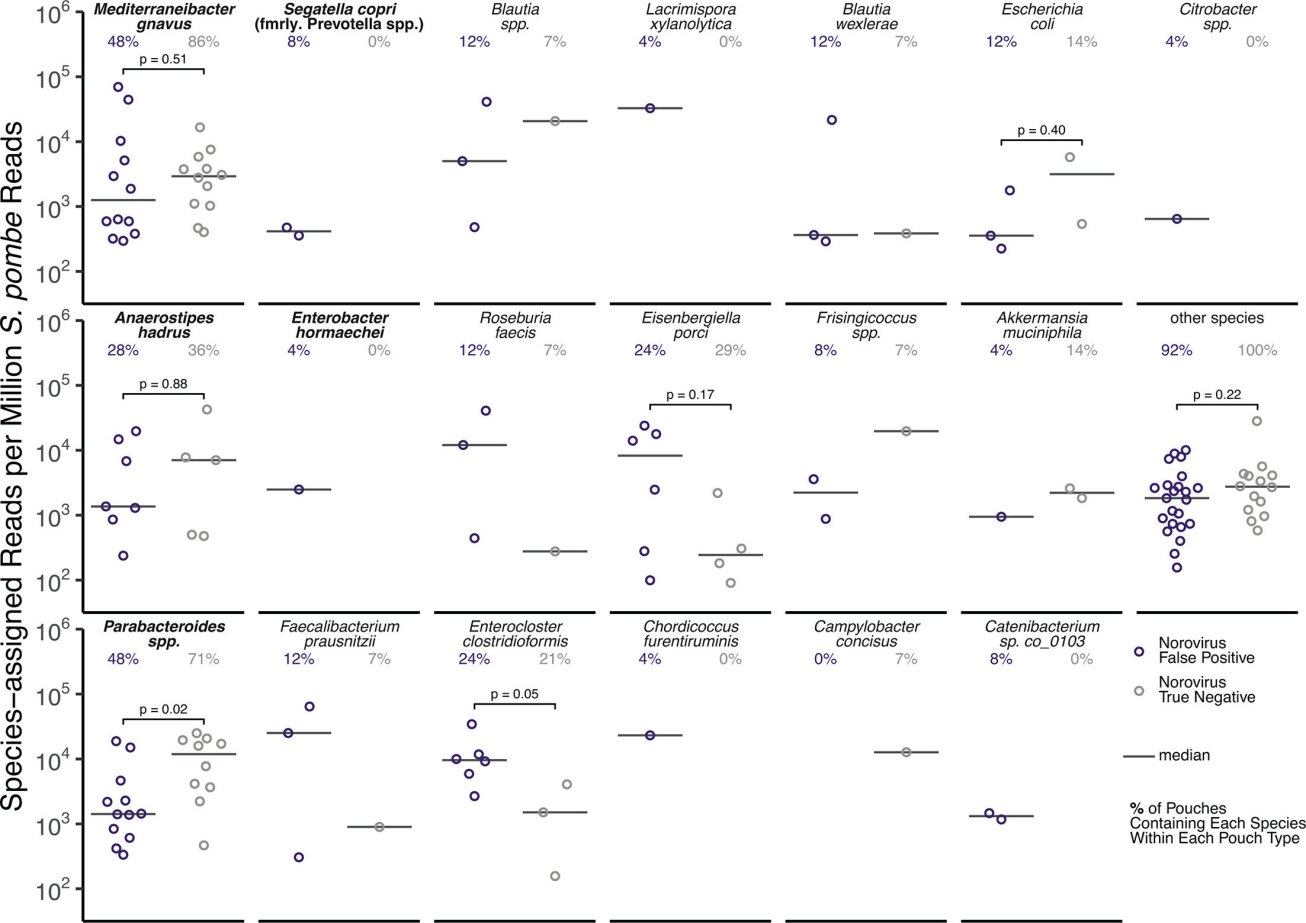
Noro-1 assay off-target amplification in norovirus false-positive pouches is heterogeneous and not attributable to a single organism. For each pouch in which the dominant species was detected (see Fig. 3), the mean *S. pombe*-normalized RPM (*n* = 2) is plotted, with norovirus false-positive pouches shown in purple and norovirus true-negative pouches shown in gray. The median RPM for each species is indicated by a horizontal bar. The percentage of norovirus false-positive and norovirus true-negative pouches from which each species was recovered is shown below each species name (in purple and gray, respectively). *P*-values from two-sided Wilcoxon rank-sum tests comparing log_10_-transformed *S. pombe*-normalized RPM between norovirus false-positive and norovirus true-negative pouches are shown for species with at least two observations in both groups. The organisms are ordered by the maximum *S. pombe*-normalized RPM observed across all norovirus false-positive pouches, with species listed in the bioMérieux IFU shown first (in bold).

To identify which species contributed most to the overall Noro-1 signal, the highest-RPM off-target species in each norovirus false-positive pouch was identified by normalized RPM and hereafter referred to as the dominant species (Fig. 3). These included all five off-target species listed in the BF-GIP IFU (Fig. 3, shown in bold), with *Mediterraneibacter gnavus*, *Anaerostipes hadrus*, and *Parabacteroides spp*. frequently detected in both false-positive and true-negative pouches (48% vs 86%, 28% vs 36%, and 48% vs 71%, false positive vs true negative, respectively; Fig. 4). For these species, median normalized RPM was higher among the true negatives than the false positives, suggesting that they are unlikely to be the sole cause of the false-positive BF-GIP results. Other species not listed in the IFU with higher median normalized RPM among false-positive compared to true-negative pouches included *Enterocloster clostridioformis* and *Eisenbergiella porci*. However, these species did not appear consistently across pouches, with each identified in no more than six pouches across both groups. Overall, these exploratory comparisons did not reach statistical significance except for *Parabacteroides* spp. (*P* = 0.02) and *Enterocloster clostridioformis* (*P* = 0.05), with several species excluded from statistical testing due to fewer than two observations in one or both groups (see Materials and Methods).

Given that no single species accounted for the off-target amplification, we hypothesized that the cumulative Noro-1 off-target burden drove the false-positive BF-GIP results. To test this, we compared the percentage of reads assigned as Noro-1 amplicons across false-positive, true-negative, and true-positive pouches (Fig. S5). The mean percentage of Noro-1 amplicons varied broadly across false-positive pouches, ranging from 0.08% to 6.83% (median 1.85%), compared to true-negative pouches (0.59%–3.75%; median 1.83%) and true-positive pouches (0.94%–1.59%; median 1.41%; Fig. S5). The percentage of Noro-1 amplicons was highly reproducible between independent library preparations across all pouch types. Given similar median levels of Noro-1 amplicons between false-positive and true-negative pouches (1.85% and 1.83%, respectively), off-target amplicon levels alone were not predictive of false-positive BF-GIP norovirus results.

To test whether off-target Noro-1 amplification was driven by specific loci in each off-target species, the Noro-1 amplicons were aligned to their respective species when a suitable reference could be identified (references with ≥95% identity were identified for amplicons from 16 of 19 dominant species listed in Fig. 3; Fig. S6). For 13 of the 16 species, amplicons aligned predominantly to a single locus within each species. In contrast, amplicons from the remaining three species aligned predominantly to two loci (*Escherichia coli*, *Lacrimispora xylanolytica*, and *Roseburia faecis*). Together, these results indicate that Noro-1 off-target amplification is driven by amplification at specific genomic loci rather than random priming. To further characterize these loci, the inferred Noro-1 primer binding sites were examined at the loci with the highest mapped amplicon count for each species (representing >10% of assigned reads), using the amplicon alignments to determine forward and reverse primer binding positions (Fig. S7). Across the loci examined, the inferred forward primer overlap ranged from 7 to 17 bases, and the inferred reverse primer overlap ranged from 4 to 16 bases. The number of loci where primers overlapped by at least 10 bases was higher for the inferred forward primer (18 of 21) compared to the reverse primer (14 of 21). Mismatches within the overlap regions ranged from 0 to 4 for both the forward and reverse primers.

To determine whether the off-target amplicons detected from deep sequencing could account for the BF-GIP instrument’s positive norovirus calls, the estimated Tm of detected off-target amplicons was compared to the Tm values and Cp values reported by the BF-GIP instrument for the Noro-1 and Noro-2 assays across false-positive and true-negative pouches (Fig. S8 and 9). Among the 25 false-positive pouches, 20 were Noro-1 positive, and 5 were Noro-2 positive by the instrument, with generally high Cp or no Cp values reported (Noro-1: 16 of 20 with reported Cps ranging from 24.0 to 30.0, 4 of 20 with no reported Cp; Noro-2: 3 of 5 with reported Cps of 30.0, 2 of 5 with no reported Cp). No false-positive pouches were positive for both Noro-1 and Noro-2 assays. For the Noro-1 assay, off-target amplicons were detected in all 20 Noro-1-positive false-positive pouches, though levels varied substantially across pouches, with many detected below 2,500 *S. pombe*-normalized RPM (Fig. S8A). In many pouches, detected amplicon Tm values overlapped with instrument-reported Tm values and fell within the estimated acceptable range, consistent with off-target amplification driving the positive call. Among the five Noro-1-negative false-positive pouches, off-target Noro-1 amplicons were detected by sequencing in all pouches, with amplicon Tm values falling within the estimated acceptable range in four of the five pouches (all except pouch 504; Fig. S8A). However, instrument-reported Tm values were absent in all except pouch 038, suggesting that off-target Noro-1 amplification was present but below the level required to trigger a positive instrument call. For the Noro-2 assay, off-target amplicons were detected in only one of five Noro-2-positive pouches, with amplicon Tm values overlapping the instrument-reported Tm in one pouch (pouch 038; Fig. S8B). Among the 20 Noro-2-negative pouches, two pouches had instrument-reported Tm values (pouch 006 and 367) with an amplicon detected in only pouch 367 with a Tm outside the estimated acceptable range (Fig. S8B).

Among the 14 true-negative pouches, off-target Noro-1 amplicons were detected in all pouches, with Tm values also falling within the estimated acceptable range except for pouch 832 (Fig. S9A). Despite the presence of these off-target amplicons, instrument-reported Tm values were present in only one pouch (pouch 526), suggesting that off-target amplification in true-negative pouches was generally below the level required to trigger a positive instrument call. No off-target Noro-2 amplicons were detected in any true-negative pouch (Fig. S9B).

**Fig 5:**
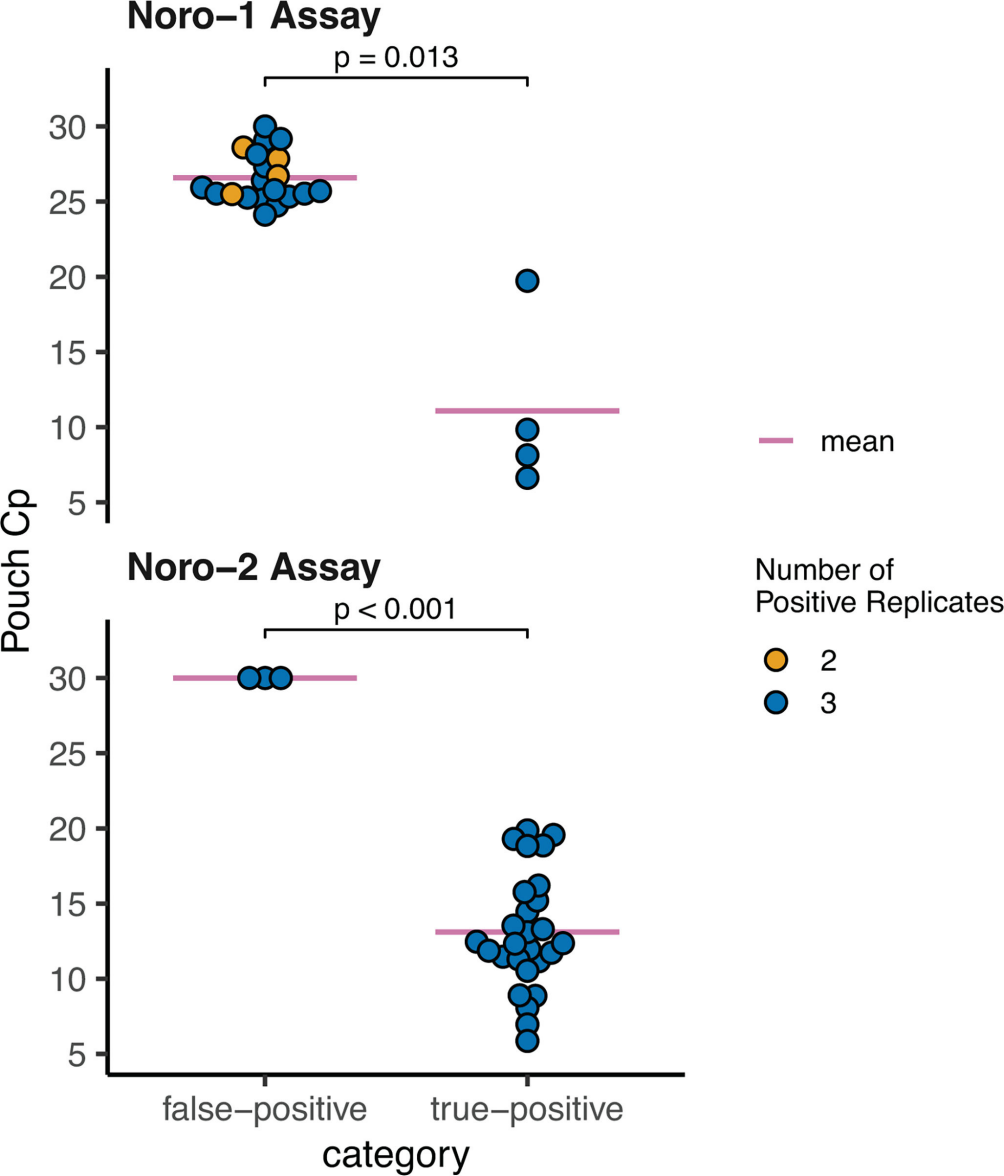
Norovirus false-positive pouches exhibit higher Cp values than true-positive pouches. Pouch Cp values reported by the BF-GIP instrument are shown for false-positive pouches and true-positive pouches for the Noro-1 (upper panel) and Noro-2 (lower panel) assays. The true-positive group includes the three true-positive pouches identified in this study and 28 additional BD MAX-confirmed norovirus-positive pouches not otherwise analyzed. Each point represents the mean Cp for a single pouch, with fill color indicating the number of instrument replicates or wells reporting a Cp value (orange: two wells and blue: three wells). The horizontal bar indicates the mean Cp for each group. *P*-values are from two-sided *t*-tests.

Given the overall high Cp values observed among norovirus false-positive pouches, we compared BioFire instrument-reported Noro-1 and Noro-2 Cp values between false-positive and true-positive pouches. To supplement the three true-positive pouches available in this data set, Cp values from 28 additional BD MAX-confirmed norovirus-positive pouches retrieved from BioFire FireWorks software were included in the comparison, for a total of 31 true-positive pouches (*n* = 4, Noro-1 positive; *n* = 27, Noro-2 positive). Two of the false-positive pouches did not have a reported Cp for either the Noro-1 or Noro-2 assay (pouches 488 and 478) and were excluded (*n* = 20 Noro-1 positive; *n* = 3 Noro-2 positive). Mean pouch Cp values (based on three FilmArray wells per assay) were significantly higher in false-positive than true-positive for both the Noro-1 assay (26.6 vs 11.1; *P* = 0.013) and the Noro-2 assay (30.0 vs 13.1; *P* < 0.001; Fig. 5).

## DISCUSSION

Although molecular multiplex panels have the potential for enhancing diagnostics and patient care through faster results and broad testing, results from our study indicate significant specificity problems in the BF-GIP Noro-1 assay, consistent with cross-reactivity documented exclusively for this assay in the BF-GIP IFU. These issues likely stem from the inherent limitations of a primer-only-based qPCR that lacks the specificity of probe-based assays and is more prone to mispriming at non-specific sites or cross-reactive genes (27).

Reported FDRs for the BF-GIP norovirus assay have ranged from 20% to 66% (9, 10, 12, 14, 28). Here, we observed a slightly higher and broader FDR of 31%–74%, likely reflecting differences in patient case mix. For example, the highest FDR was observed at the specialized cancer center. In cancer patients, in whom chemotherapy-induced diarrhea is common (29), the cause of diarrhea is more likely to be non-infectious, reducing the pre-test probability of norovirus infection and increasing the FDR. Beyond differences in pre-test probability, chemotherapy, antibiotic exposure, and mucosal injury can alter gut microbiome composition in immunocompromised patients, potentially enriching cross-reactive taxa and increasing off-target template abundance in stool. Additional epidemiological factors such as winter seasonality and the presence of vomiting can significantly affect the prior probability and thus the positive predictive value for norovirus testing.

Given the scale of BF-GIP deployment, modest reductions in specificity can produce a substantial false-positive burden. For example, the decline in NPA from 98.8% reported at FDA approval (14) to 96.5% in the subsequent postmarket performance follow-up study (6) would approximately triple the annual false-positive burden. We estimate 21,804–26,496 false positives per year at an NPA of 98.8%, rising to 63,595–77,280 at 96.5% (see Materials and Methods). Settings with lower norovirus prevalence or lower assay specificity, as has been reported in recent clinical use (12), would be expected to result in higher false-positive burdens.

To investigate the molecular basis of these false-positive results, we developed a novel workflow to extract and sequence BF-GIP pouch amplicons to identify off-target amplification. To accurately bin off-target sequences, we had to determine the specific primer sequences used in the assay. Sequencing confirmed that off-target amplification was almost exclusively attributable to the Noro-1 assay, with 78 off-target species identified compared to only 2 for the Noro-2 assay. All cross-reactive organisms listed in the BF-GIP IFU are represented among the detected off-target amplicons (Fig. 3). Amplicons from 16 of 19 dominant off-target species mapped to discrete genomic loci with partial overlap between the off-target amplicon and the inferred Noro-1 primer sequences (Fig. S6 and S7), consistent with mispriming at relatively discrete sites rather than random off-target amplification.

Because off-target amplification was detected in both false-positive and true-negative pouches at overlapping levels, the basis for the false-positive results remained unclear (Fig. 3). The BF-GIP uses Tm analysis to confirm positivity, so a false-positive result requires not only off-target amplification but also an off-target amplicon Tm sufficiently close to that expected for the norovirus amplicon. Although detected amplicons in most false-positive pouches fell within the acceptable Tm range, this also held true for the true-negative pouches (Fig. S89). Thus, based on sequencing results alone, it remains unclear why off-target amplification resulted in a positive call by the BioFire instrument.

There are several limitations to consider for this Tm analysis. First, since the acceptable Tm range is not publicly disclosed, ranges were inferred from Noro-1 and Noro-2 positive assay results exported from the FireWorks software and may not be strictly accurate. Second, the exact reaction conditions are unknown (e.g., monovalent and divalent ion concentrations) and were estimated by selecting conditions that yielded a theoretical Tm for the expected Noro-1 and Noro-2 amplicons closest to the midpoint of the inferred acceptable Tm range. Despite these limitations, in many cases, our estimated amplicon Tms were concordant with instrument-reported Tms (e.g., 19 out of 25 false-positive pouches in the Noro-1 assay had at least one amplicon detected that overlapped with reported Tms).

The relatively consistent recovery of the RNA process control (*S. pombe* assay) suggests that our amplicon extraction methodology recovered a representative sample across pouches. However, in many cases, we did not detect an amplicon at high levels that corresponded to one of the reported Tms (e.g., pouch 006 in Fig. S8). In some of these cases, no Cp was reported, or the Cp was relatively high, suggesting that reported Tms may have derived from weakly amplified amplicons. A major limitation of this analysis is the assumption that sequencing results are semiquantitative, but our results are likely impacted by incomplete sampling of the amplicons from the closed pouch or PCR bias that might skew the abundance of specific amplicons. Equally, the Cp values reported by the instrument are likely not fully quantitative, given the primer mismatch to templates and the nested amplification approach. Furthermore, we only sequenced BF-GIP amplicons in bulk and did not spatially resolve amplicons by target wells in the array, as this would be laborious and impractical. In addition, our study was limited by the absence of discrete spike-in oligonucleotides, which precluded normalization of sequencing data and the estimation of absolute amplicon abundance.

Both Matic et al. and Caza et al. have investigated the use of melt curve analysis to confirm norovirus results. However, both groups reported finding true-positive results with atypical melting curves and false-positive results with typical melting curves (9, 10). Melt curves were not examined in this study. Another study found that norovirus false-positive Cp values were higher compared to true-positive results (11), and we confirmed this finding (Fig. 5). This difference suggests that Cp values may help prioritize which BF-GIP norovirus-positive results are sent for confirmatory testing, though the optimal Cp threshold and its impact on the sensitivity and specificity of such a reflex algorithm would require prospective validation with larger data sets. A further consideration is that chronic norovirus infection can result in prolonged low-level shedding (30). These low-level infections may fall between the LoDs of the BF-GIP and BD MAX, with the BF-GIP LoD of 1 × 10⁴ RNA copies/mL (6) being substantially more sensitive than the BD MAX LoD of 4.71 × 10⁶ copies/mL (31). Thus, some true low-level positives could be incorrectly classified as false positives by confirmatory testing, reducing the sensitivity of the reflex algorithm. Finally, this approach would also require installation and review of BioFire FireWorks software, which may not be available at all sites.

Notably, 8 of 25 false-positive pouches (32%) were positive for at least one additional panel target, with similar rates of co-detection observed among all true-positive pouches (10 of 31 true-positive pouches, including sequenced and supplemental; 32%; Table S3). Whether co-detection influences false-positive rates, potentially through co-infection-driven shifts in gut microbiota favoring cross-reactive species, may warrant further investigation. However, one small study (*n* = 50) found that the number of additional positive panel targets did not affect the likelihood of a negative confirmatory result (10).

Although we observed clear evidence that the Noro-1 assay primers appear to frequently misprime and generate off-target amplicons, we could not demonstrate a clear concordance between detection of these off-target amplicons by sequencing and the instrument-reported positive results. Regardless, the number of potential cross-reactive species appears to be substantially greater than that listed in the updated BioFire IFU. In healthcare settings, norovirus positivity frequently triggers isolation and cohorting decisions, and false-positive results may lead to unnecessary contact precautions, inappropriate ward placement, disruption to patient flow, and potential anchoring on the wrong diagnosis. Given that norovirus is the leading cause of acute gastroenteritis and foodborne illness worldwide (32), these inaccuracies in norovirus testing raise concerns for patient care and underscore the critical need for more reliable testing strategies, including redesign of this assay and continued confirmatory testing.

## Data Availability

In order to not publish proprietary BF-GIP primer sequences, we have not deposited sequencing reads into NCBI Sequence Read Archive. Data from the analysis are available from the authors upon reasonable request.

## SUPPLEMENTAL MATERIAL

Figure S1 jcm.00745-26-s0001.pdf Flow chart.

Figure S2 jcm.00745-26-s0002.pdf Amplicon assignments.

Figure S3 jcm.00745-26-s0003.pdf Off-target.

Figure S4 jcm.00745-26-s0004.pdf *S. pombe* amplicons.

Figure S5 jcm.00745-26-s0005.pdf Noro-1 assay amplicons.

Figure S6 jcm.00745-26-s0006.pdf Noro-1 off-target amplicons.

Figure S7 jcm.00745-26-s0007.pdf Primer figure.

Figure S8 jcm.00745-26-s0008.pdf False positive Tm figure.

Figure S9 jcm.00745-26-s0009.pdf True negative Tm figure.

Supplemental tables jcm.00745-26-s0010.xlsx Tables S1 to S3.

ASM does not own the copyrights to Supplemental Material that may be linked to, or accessed through, an article. The authors have granted ASM a non-exclusive, world-wide license to publish the Supplemental Material files. Please contact the corresponding author directly for reuse.
