# Supplementary material for: Molecular characterization of norovirus false-positive results on the BioFire FilmArray gastrointestinal panel
Source: J Clin Microbiol. 2026 Jul 27;64(8):e00745-26. doi: 10.1128/jcm.00745-26 (PMC13463817; doi:10.1128/jcm.00745-26)
Supplement: Supplementary file 1 [file jcm.00745-26-s0001.pdf]

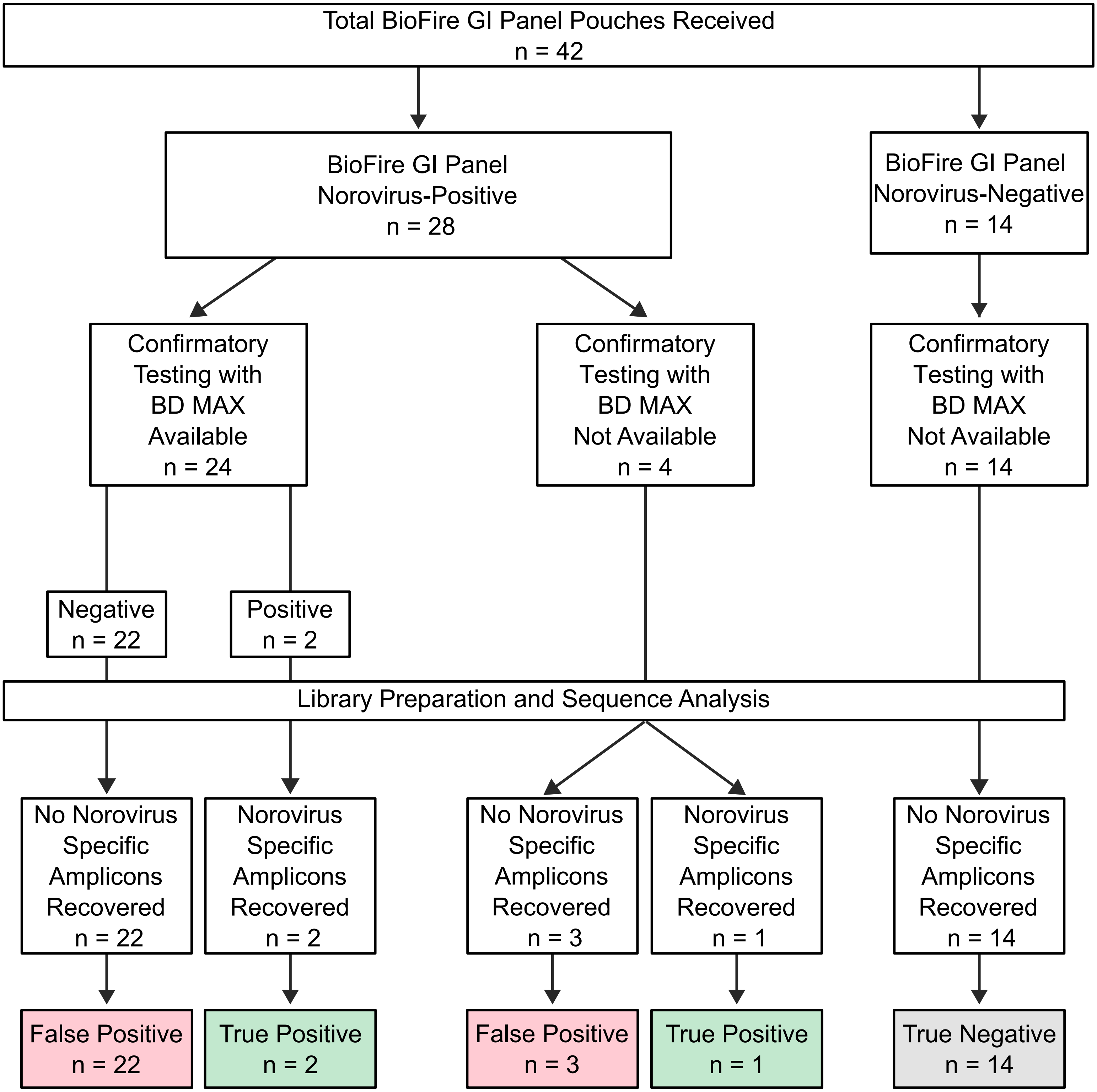

Figure S1. BF-GIP pouch classification scheme using BD MAX confirmatory testing and BLASTn identification of sequenced amplicons. A total of 42 BF-GIP pouches were received, with 28 reported norovirus-positive by the BioFire instrument and 14 reported norovirus-negative. BD MAX confirmatory testing was available for 24 of the 28 BioFire-reported norovirus-positive pouches but was not available for the remaining 4 norovirus-positive or any of the 14 norovirus-negative pouches. For pouches without BD MAX confirmatory testing, classification was based on sequencing results. BioFire-reported norovirus-positive pouches with norovirus-specific amplicons identified among Noro-1 or Noro-2 assay filtered reads were classified as norovirus-true-positive, and those without were classified as norovirus-false-positive. BioFire-reported norovirus-negative pouches without norovirus-specific amplicons were classified as norovirus-true-negative. Of the 24 BD MAX-confirmed pouches, 22 were norovirus-false-positive and 2 were norovirus-true-positive. Among the remaining four BioFire-reported norovirus-positive pouches, three had no norovirus-specific amplicons identified and were classified as norovirus-false-positive, and one had norovirus-specific amplicons identified and was classified as norovirus-true-positive. As expected, all 14 BioFire-reported norovirus-negative pouches lacked norovirus-specific amplicons and were classified as norovirus-true-negative. In total, 25 norovirus-false-positive, 3 norovirus-true-positive, and 14 norovirus-true-negative pouches were identified.
