# Supplementary material for: Molecular characterization of norovirus false-positive results on the BioFire FilmArray gastrointestinal panel
Source: J Clin Microbiol. 2026 Jul 27;64(8):e00745-26. doi: 10.1128/jcm.00745-26 (PMC13463817; doi:10.1128/jcm.00745-26)
Supplement: Supplementary file 2 [file jcm.00745-26-s0002.pdf]

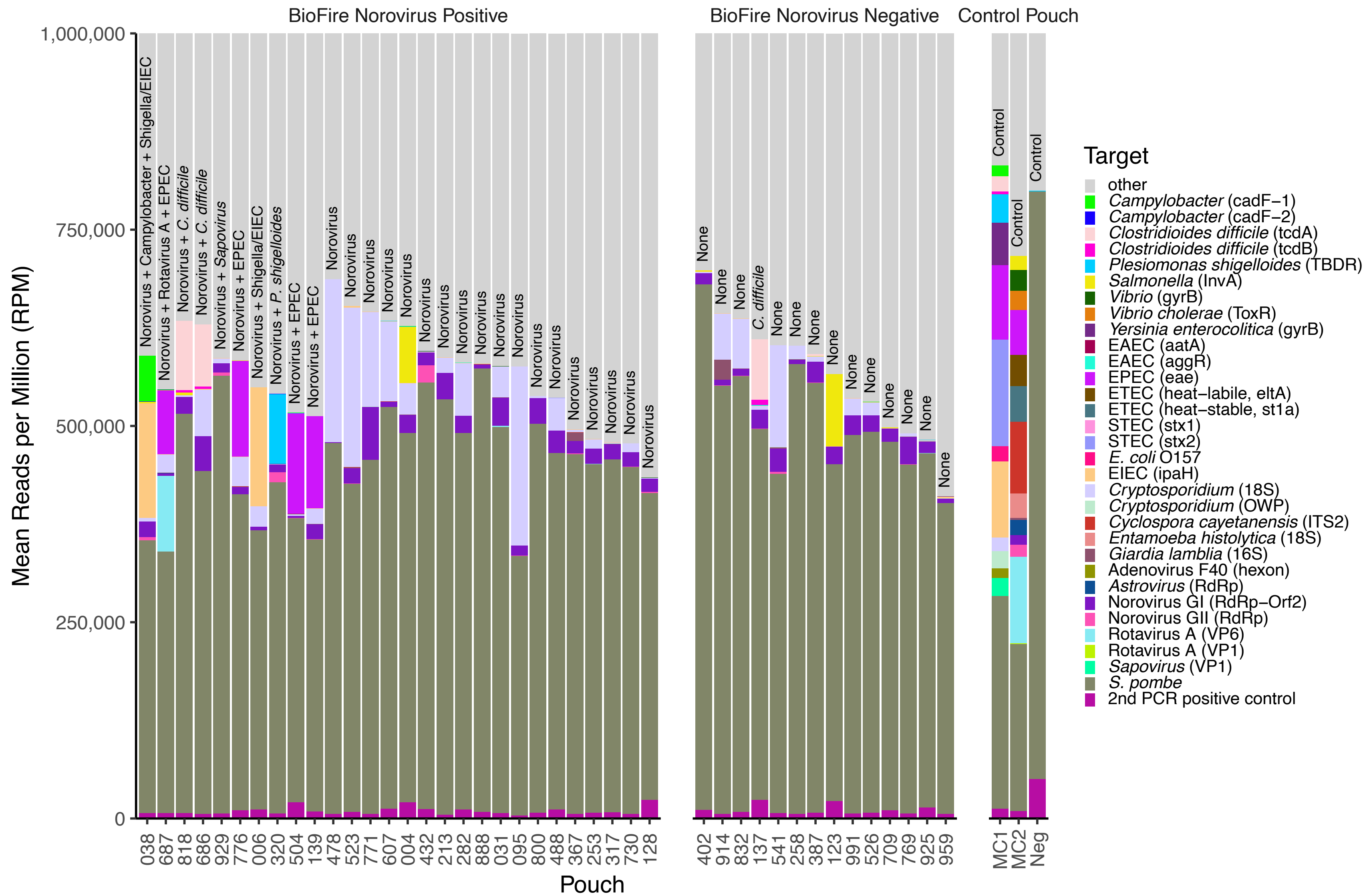

Figure S2. BF-GIP pouch amplicon assignments based on inferred primer sequences across BioFire norovirus-positive, BioFire norovirus-negative, and control pouches. BF-GIP pouch amplicons from 28 BioFire-reported norovirus-positive, 14 BioFire-reported norovirus-negative, and 3 control pouches (MC1, MC2, and negative control) were sequenced and reads were assigned to specific BF-GIP assays using primer sequences inferred from analysis of MC1 and MC2 sequencing data. The mean RPM across two library preparations is shown for each pouch, with each color representing a different BF-GIP assay target. The BioFire-reported result for each pouch is indicated above each bar. Within the norovirus-positive group, pouches are ordered first by the number of BioFire-reported targets (descending), then by total RPM excluding the unassigned reads (other). Norovirus-negative pouches are ordered by total RPM excluding the unassigned reads (other). Control pouches are shown in the order MC1, MC2, and negative control. The *S. pombe* target corresponds to the internal RNA process control, which monitors all steps in the BioFire assay, and the 2nd PCR positive target corresponds to the second-stage PCR control, which monitors the final PCR amplification step. As expected, both controls are present in all pouches. We noted that the *Cryptosporidium* (18S) primers were frequently detected, but examination of the estimated Tm values of the associated amplicons revealed they were significantly higher than the expected *Cryptosporidium* 18S locus, explaining the absence of a positive call for *Cryptosporidium* among these pouches.
