# Supplementary material for: Molecular characterization of norovirus false-positive results on the BioFire FilmArray gastrointestinal panel
Source: J Clin Microbiol. 2026 Jul 27;64(8):e00745-26. doi: 10.1128/jcm.00745-26 (PMC13463817; doi:10.1128/jcm.00745-26)
Supplement: Supplementary file 3 [file jcm.00745-26-s0003.pdf]

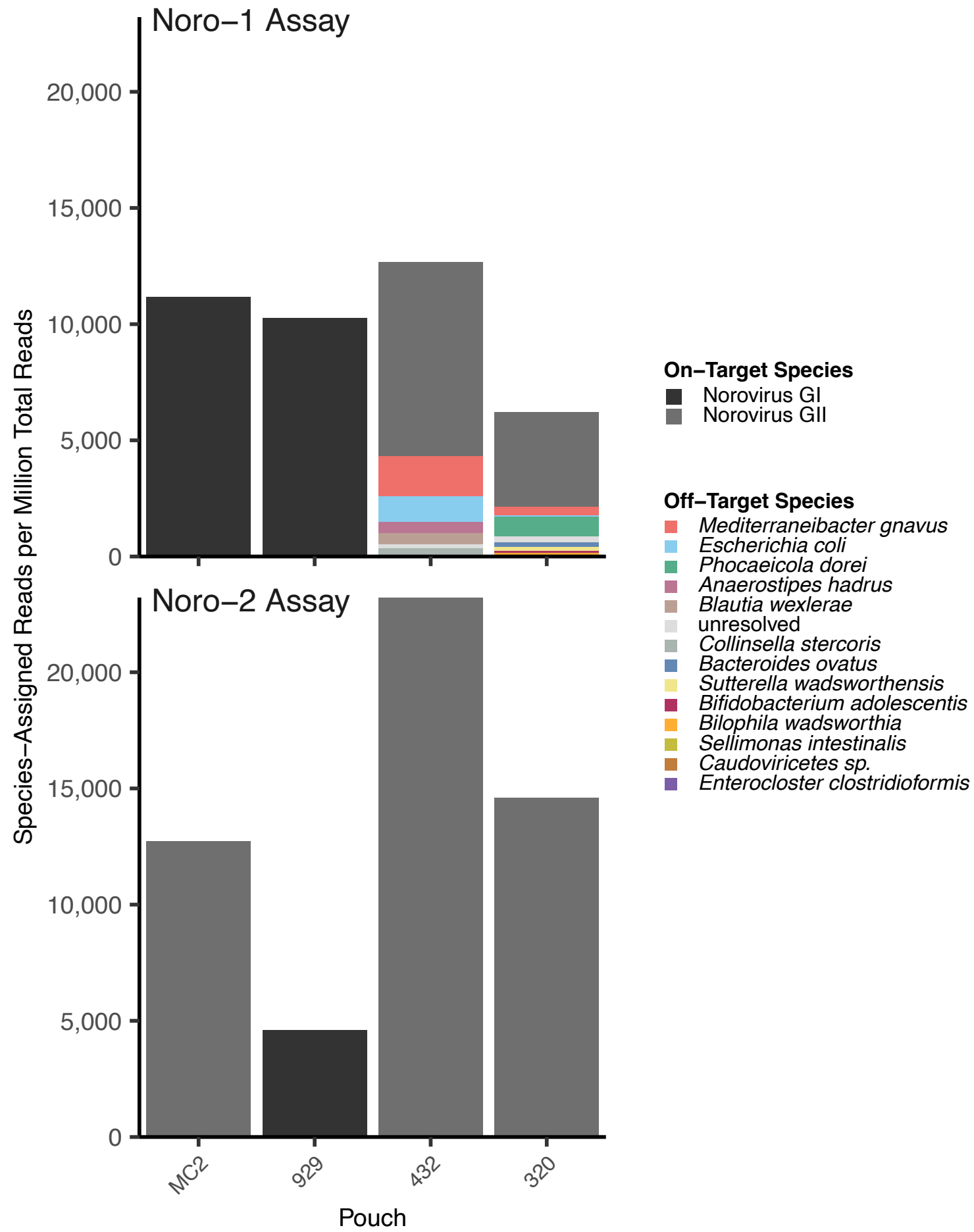

Figure S3. Off-target amplification was limited to the Noro-1 assay and was not observed in all norovirus true-positive pouches. Reads assigned to the Noro-1 (upper panel) and Noro-2 (lower panel) assay from BF-GIP testing of the MC2 control pouch and three norovirus-true-positive clinical pouches (929, 432, and 320) were analyzed by BLASTn against the NCBI Core nt database for species identification. The mean RPM of reads assigned to each species across two library preparations is shown for each pouch. On-target species (Norovirus GI and Norovirus GII) are shown in black and grey, respectively, and off-target species in other colors as indicated in the legend. Unresolved reads indicate cases where BLASTn results could not be resolved to a single species. Off-target amplicons were detected exclusively among the Noro-1 assay assigned reads in pouches 432 and 320, with no off-target amplicons detected in the Noro-2 assay assigned reads across any pouch. Although only Norovirus GI was detected in pouch 929 and only Norovirus GII in pouches 432 and 320, norovirus amplicons from all three pouches were identified in both Noro-1 and Noro-2 assay assigned reads.
