# Supplementary material for: Molecular characterization of norovirus false-positive results on the BioFire FilmArray gastrointestinal panel
Source: J Clin Microbiol. 2026 Jul 27;64(8):e00745-26. doi: 10.1128/jcm.00745-26 (PMC13463817; doi:10.1128/jcm.00745-26)
Supplement: Supplementary file 4 [file jcm.00745-26-s0004.pdf]

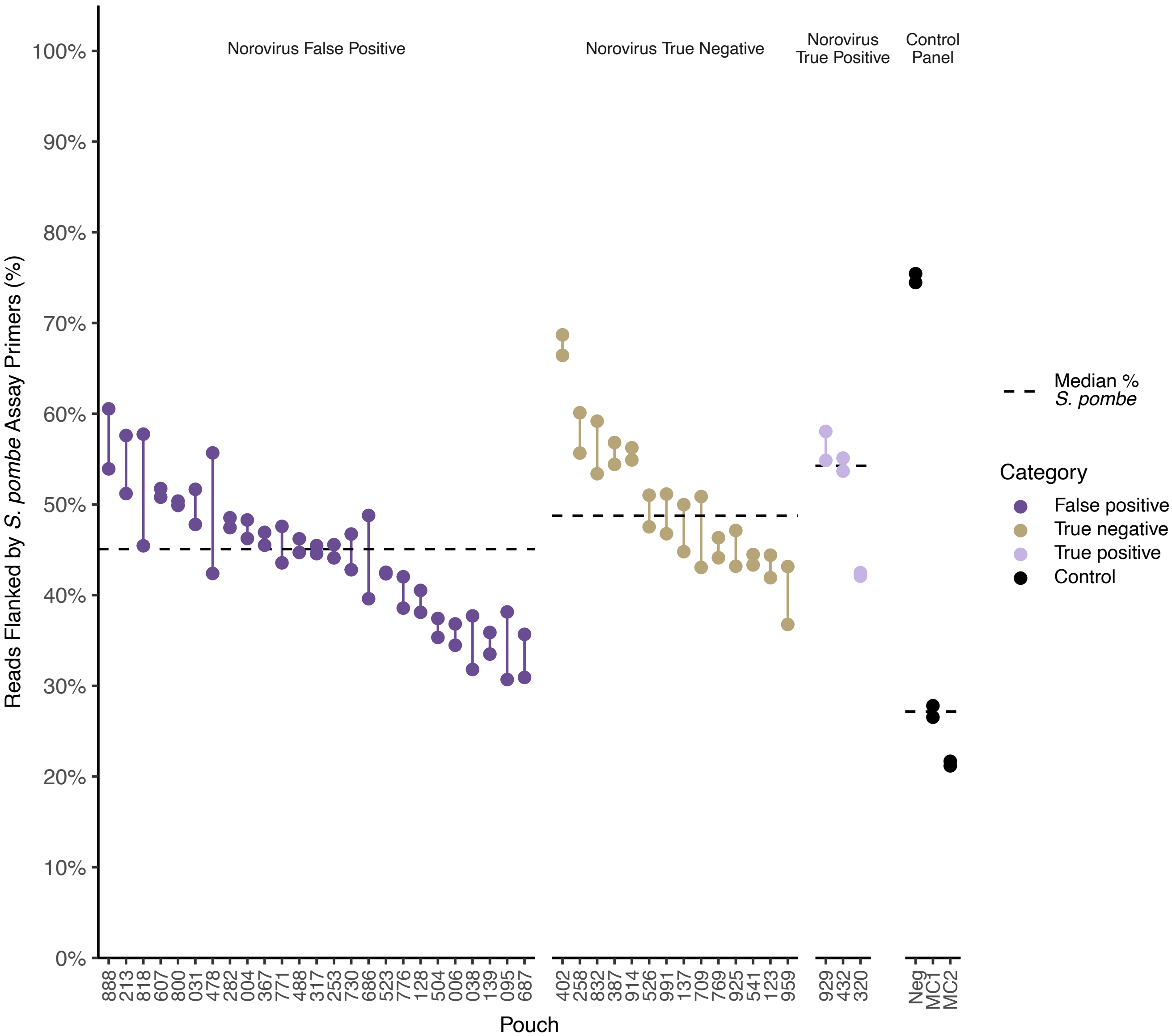

Figure S4. Recovery of *S. pombe* amplicons is comparable across norovirus-false-positive, norovirus-true-negative, and norovirus-true-positive BF-GIP pouches. The percent of merged reads assigned to the *S. pombe* assay using the inferred primers is shown for each library preparation (n = 2), color-coded by classification, with the two library preparations per pouch connected by a line. Pouches are shown separated by classification and ordered from highest to lowest *S. pombe* percentage. Within each classification, the median percent of reads assigned to the *S. pombe* assay is shown with a dashed line.
