# Supplementary material for: Molecular characterization of norovirus false-positive results on the BioFire FilmArray gastrointestinal panel
Source: J Clin Microbiol. 2026 Jul 27;64(8):e00745-26. doi: 10.1128/jcm.00745-26 (PMC13463817; doi:10.1128/jcm.00745-26)
Supplement: Supplementary file 5 [file jcm.00745-26-s0005.pdf]

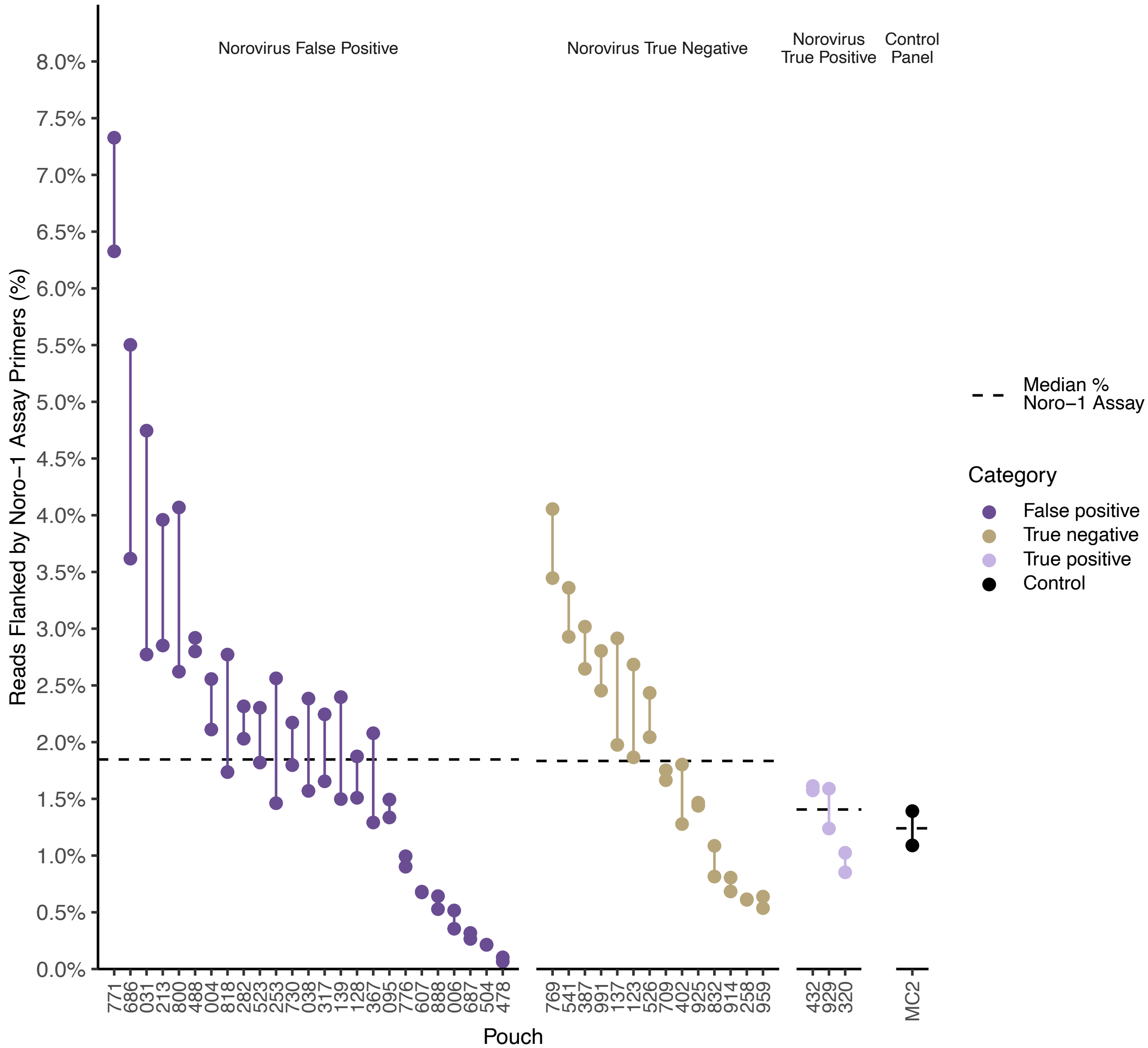

Figure S5. Percentage of reads assigned to the Noro-1 assay overlaps between norovirus-false-positive and norovirus-true-negative pouches. The percent of merged reads assigned to the Noro-1 assay using the inferred primers is shown for each library preparation (n = 2), color-coded by classification, with the two library preparations per pouch connected by a line. Pouches are shown separated by classification and ordered from highest to lowest percentage of reads assigned to the Noro-1 assay. Within each classification, the median percent of reads assigned to the Noro-1 assay is shown with a dashed line.
