# Supplementary material for: Molecular characterization of norovirus false-positive results on the BioFire FilmArray gastrointestinal panel
Source: J Clin Microbiol. 2026 Jul 27;64(8):e00745-26. doi: 10.1128/jcm.00745-26 (PMC13463817; doi:10.1128/jcm.00745-26)
Supplement: Supplementary file 6 [file jcm.00745-26-s0006.pdf]

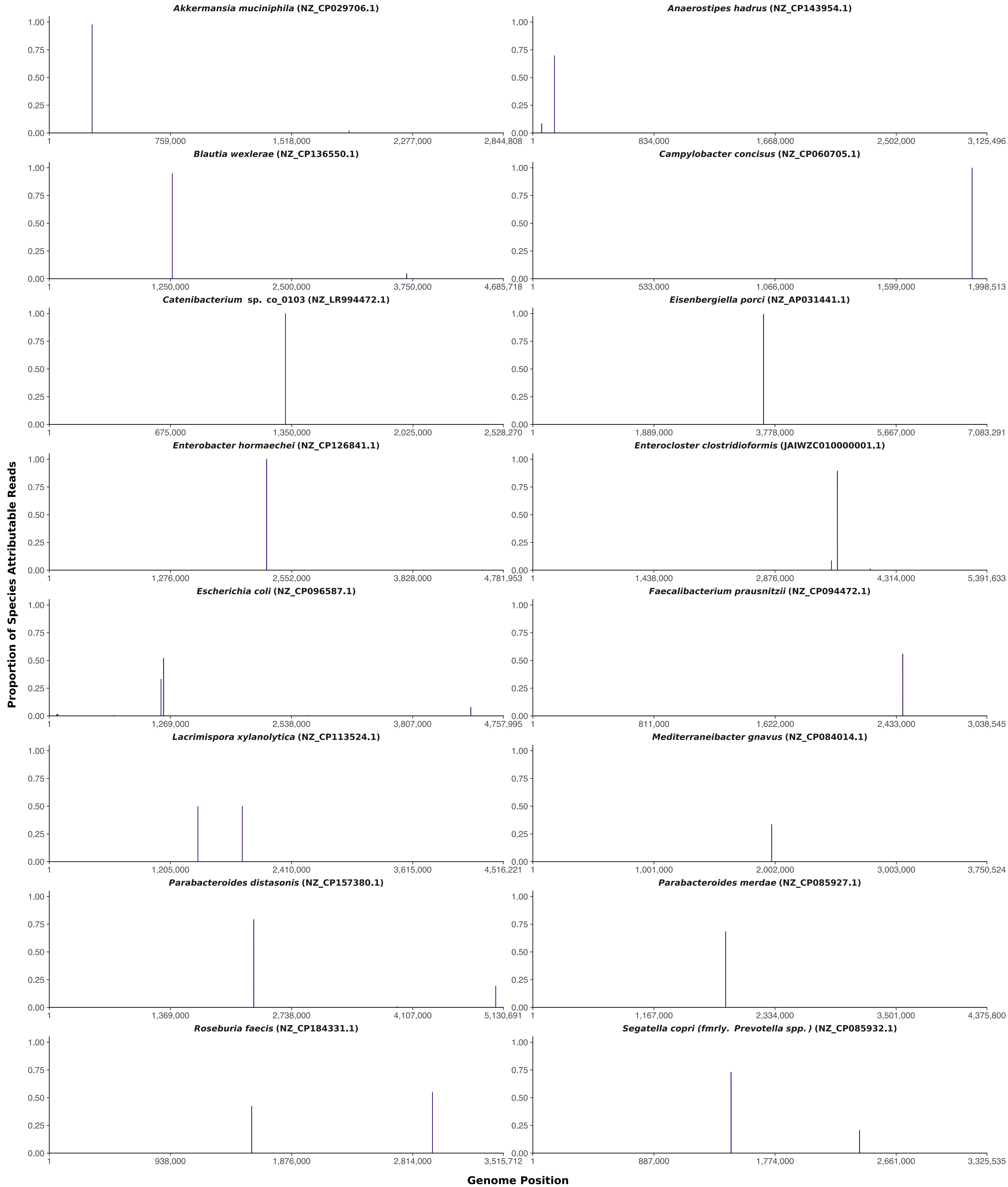


**Figure S6. Noro-1 off-target amplicons arise from a limited number of discrete genomic loci within each cross-reactive species.** All reads attributable to each of the identifiable species in Figure 3 were mapped to corresponding genome references and base-by-base alignment counts plotted as a proportion of total reads from each species. The majority of amplicons mapped to one or two sites per species, demonstrating that off-target amplification occurs at discrete genomic loci rather than randomly across the genome. Bars do not sum to 1 as non-mappable amplicons were counted in the totals. *Chordicoccus furentiruminis* has been omitted as BLASTn results with at least 95% identity were not obtained.
