# Supplementary material for: Molecular characterization of norovirus false-positive results on the BioFire FilmArray gastrointestinal panel
Source: J Clin Microbiol. 2026 Jul 27;64(8):e00745-26. doi: 10.1128/jcm.00745-26 (PMC13463817; doi:10.1128/jcm.00745-26)
Supplement: Supplementary file 7 [file jcm.00745-26-s0007.pdf]

Species	Accession	Reference Start	Reference End	Fwd Overlap	Fwd Mismatches	Rev Overlap	Rev Mismatches
Parabacteroides distasonis	CP103256.1	4932472	4932533	10	1	16	3
Enterobacter hormaechei	CP129250.1	3729291	3729357	17	2	13	1
Anaerostipes hadrus	FP929061.1	2173318	2173393	15	1	15	2
Segatella copri (fmrly. *Prevotella* spp.)	CP134816.1	2299565	2299635	10	1	8	0
Lacrimispora xylanolytica	CP113524.1	1919779	1919855	11	0	11	2
Eisenbergiella porci	AP031441.1	3598493	3598571	12	1	10	2
Akkermansia muciniphila	CP029706.1	268269	268354	9	0	14	3
Roseburia faecis	AP042441.1	2709462	2709554	14	2	5	1
Campylobacter concisus	CP147775.1	1170439	1170556	15	2	15	1
Parabacteroides merdae	CP081901.1	3764008	3764120	12	1	12	1
Blautia wexlerae	CP098409.1	2005768	2005890	17	4	5	0
Mediterraneibacter gnavus	CP111086.1	693434	693561	10	0	11	2
Catenibacterium sp. co_0103	LR994472.1	1314906	1315044	15	2	6	0
Parabacteroides distasonis	CP103178.1	2253202	2253349	9	0	14	3
Roseburia faecis	CP184331.1	2966414	2966566	13	0	5	0
Lacrimispora xylanolytica	CP113524.1	1478563	1478729	14	2	13	2
Escherichia coli	CP074928.1	4276054	4276241	7	0	13	2
Faecalibacterium prausnitzii	CP117963.1	1065323	1065514	16	2	8	0
Segatella copri (fmrly. *Prevotella* spp.)	CP152348.1	1909687	1909880	14	3	4	0
Enterocloster clostridioformis	JBWJAI010000158.1	1257	1466	11	0	12	1
Escherichia coli	CP057535.1	1181268	1181501	12	1	10	0

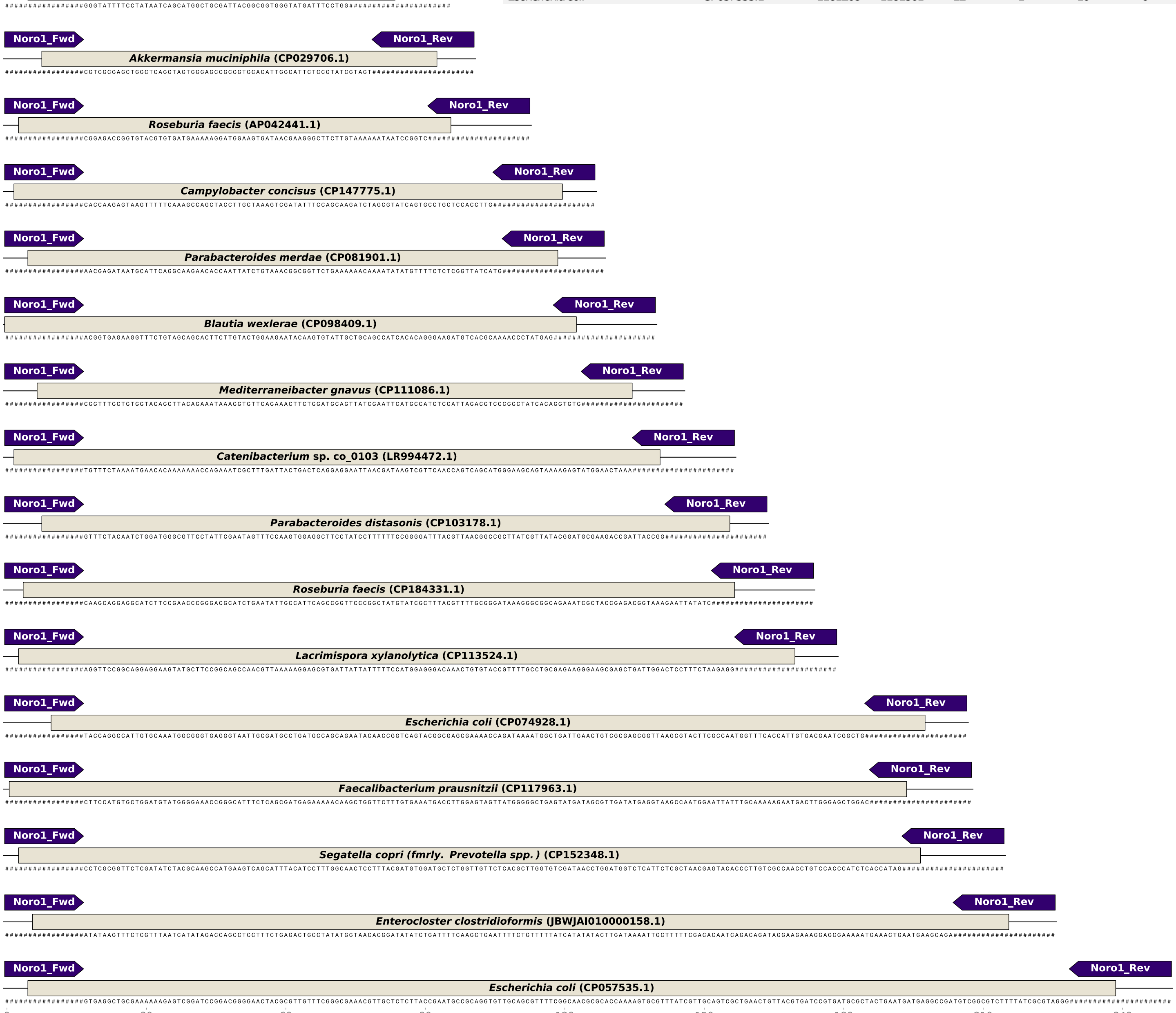

Figure S7. Variable overlap of inferred Noro-1 primers with bacterial reference sequences at off-target loci. The most abundant member of each cluster of amplicons representing 10% or more of reads for a respective species is annotated (gold) with the region corresponding to its highest-scoring BLASTn result. The inferred Noro1_Fwd and Noro1_Rev primers (purple) show varying degrees of overlap with each sequence, suggesting a high level of variability in the sites enabling off-target annealing and amplification. Bases corresponding to the primer sequences have been replaced with hashes. The inset table shows the total number of bases and mismatches in the primer-overlap region of each amplicon compared to the highest-scoring BLAST result and the coordinates of each amplicon within the reference. *Chordicoccus furentiruminis* has been omitted as BLASTn results with at least 95% identity were not obtained.
