# Supplementary material for: Molecular characterization of norovirus false-positive results on the BioFire FilmArray gastrointestinal panel
Source: J Clin Microbiol. 2026 Jul 27;64(8):e00745-26. doi: 10.1128/jcm.00745-26 (PMC13463817; doi:10.1128/jcm.00745-26)
Supplement: Supplementary file 8 [file jcm.00745-26-s0008.pdf]

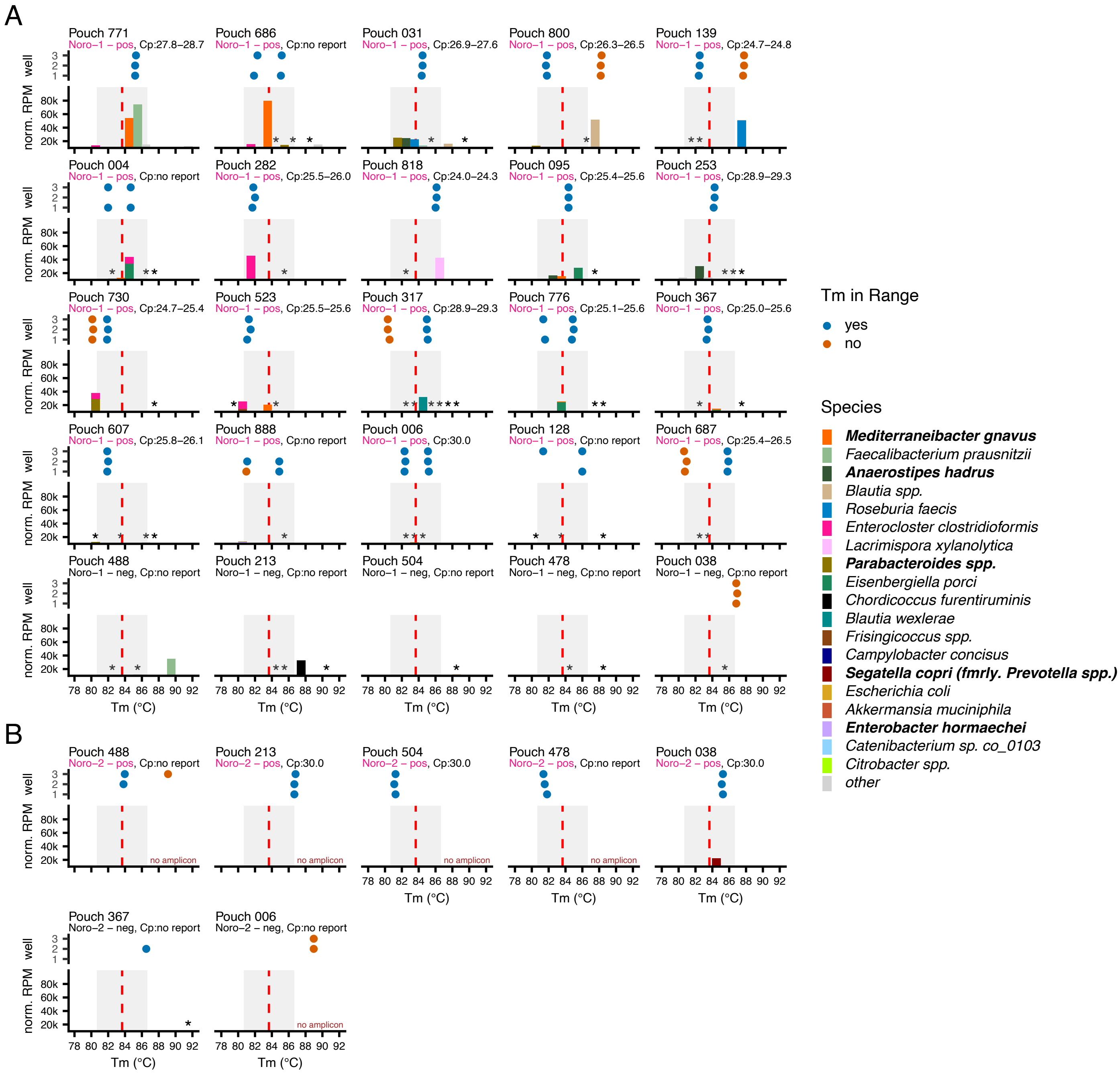

Figure S8. Comparison of off-target amplicon Tm values with BF-GIP instrument-reported Tm values in norovirus-false-positive pouches. For each norovirus-false-positive pouch, the upper panel shows the Tm values reported across the three BF-GIP FilmArray wells (wells 1–3) along with the Noro-1 or Noro-2 assay result (positive results are highlighted in magenta colored text) and Cp range. Fill color of the well Tm points indicates whether the reported Tm was within (blue) or outside (orange) the estimated acceptable Tm range (grey shaded box in lower panel). The lower panel shows the *S. pombe*-normalized RPM of off-target amplicons detected by sequencing plotted by the estimated amplicon Tm. "No amplicon" indicates no amplicons exceeding the minimum read count threshold were detected. Asterisks (*) indicate the position of amplicons detected below 2,500 *S. pombe*-normalized RPM. Fill color of the bars indicates species identity. The red dashed line indicates the midpoint of the acceptable Tm range. Panel A shows Noro-1 assay results and Panel B shows Noro-2 assay results. Pouches are ordered first by BF-GIP Noro-1 or Noro-2 result (positive then negative), then by decreasing total Noro-1 off-target RPM. Only pouches with detectable sequenced amplicons, BioFire instrument-reported Tm or Cp values, or both are shown.
