# Supplementary material for: Molecular characterization of norovirus false-positive results on the BioFire FilmArray gastrointestinal panel
Source: J Clin Microbiol. 2026 Jul 27;64(8):e00745-26. doi: 10.1128/jcm.00745-26 (PMC13463817; doi:10.1128/jcm.00745-26)
Supplement: Supplementary file 9 [file jcm.00745-26-s0009.pdf]

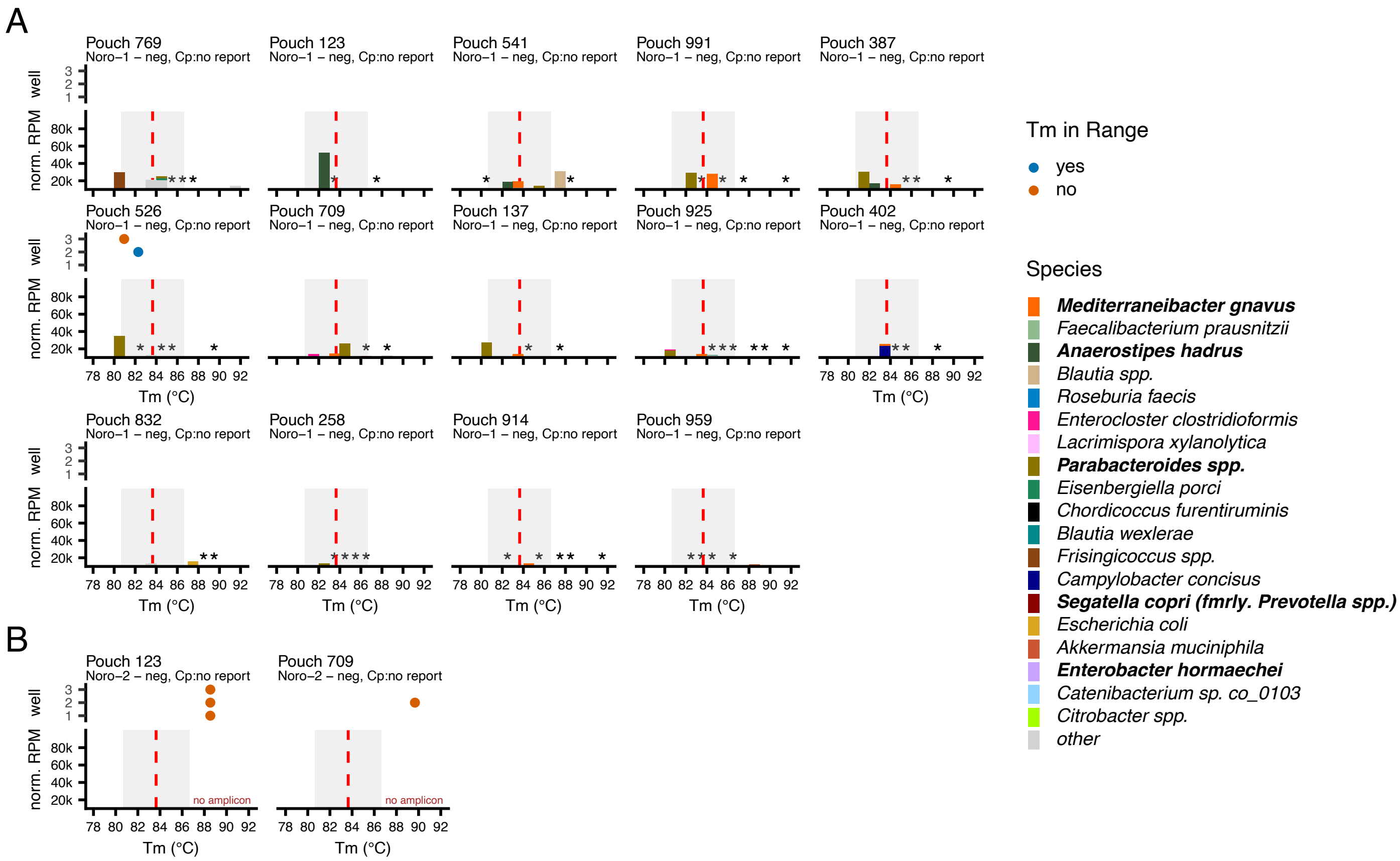

Figure S9. Comparison of off-target amplicon Tm values with BF-GIP instrument-reported Tm values in norovirus-true-negative pouches. For each norovirus-true-negative pouch, the upper panel shows the Tm values reported across the three BF-GIP FilmArray wells (wells 1–3) along with the Noro-1 or Noro-2 assay result and Cp range. Fill color of the replicate Tm points indicates whether the reported Tm was within (blue) or outside (orange) the estimated acceptable Tm range (grey shaded box in lower panel). The lower panel shows the *S. pombe*-normalized RPM of off-target amplicons detected by sequencing plotted by the estimated amplicon Tm. "No amplicon" indicates no amplicons exceeding the minimum read count threshold were detected. Asterisks (*) indicate the position of amplicons detected below 2,500 *S. pombe*-normalized RPM. Fill color of the bars indicates species identity. The red dashed line indicates the midpoint of the acceptable Tm range. Panel A shows Noro-1 assay results and Panel B shows Noro-2 assay results. Pouches are ordered by decreasing total Noro-1 off-target RPM. Only pouches with detectable sequenced amplicons, BioFire instrument-reported Tm or Cp values, or both are shown.
